# The optimal delivery time and order quantity in an oligopoly market with time-sensitive customers

**DOI:** 10.1371/journal.pone.0225436

**Published:** 2019-12-03

**Authors:** Haijiao Li, Weijin Xu, Kuan Yang

**Affiliations:** 1 School of Business Adminstration, Hunan University, Changsha, Hunan, China; 2 Supply Chain and Logistics Optimization Research Central, University of Windsor, Windsor, Ontario, Canada; Shandong University of Science and Technology, CHINA

## Abstract

With the development of e-commerce, delivery time is regarded as a key competitive advantage in an oligopoly market, as shortening the delivery time can stimulate demand for products. Many firms adopt a variety of strategies to shorten delivery time, and holding sufficient inventory is reported as an effective way. This study integrates a market share attraction model based on delivery time competition with the traditional inventory model to determine the optimal delivery time and order quantity. With the use of supermodular game method, we investigate the effect of changes in marketing and operations factors on the equilibrium delivery time and order quantity in non-dominated and dominated oligopolistic markets. The results reveal that the equilibrium delivery time and order quantity exhibit a directional response to changes in marketing and operations factors, and the response differs between the non-dominated oligopoly and the dominated oligopoly. Furthermore, under a cooperative oligopolistic market with asymmetry, it is beneficial for the firms with high competitive strength to adopt the delivery time strategy, but it fails to do so for the firm with the low competitive strength. Lastly, numerical analysis suggests that marketing factors play a more important role in affecting equilibrium measures than operations factors.

## Introduction

Under an increasingly fierce global competition, delivery time for ordered products to reach customers has become an increasingly important strategic instrument to gain competitive advantage [1–3]. Many firms are quite willing to announce a guarantee of their maximum service delivery time to attract customers’ purchase decisions [4]. For instance, Federal Express, an American international courier delivery service company, is widely advertised for its next-day shipping service for customers [5, 6]. Modern ecommerce retailers, such as Amazon, Walmart and BestBuy, regard delivery time as a key tool by providing a promised delivery time of products for customers [6–8]. According to a survey conducted by Dotcom Distribution, 87% of online shoppers identified shipping speed as a key factor in the purchase decision (mhlnews.com). In addition, business logistics firms provide dedicated facilities for time-sensitive customers with promises to ship the ordered product to clients within 24 hours [9]. JD Logistics, which is affiliated with JD.com, offers “211 limited time” service based on seven logistics centres throughout China. This service guarantees completing an order on the same day if the customer submits a spot order to JD.com before 11 a.m.; otherwise, one can still receive the product before 3 p.m. the next day [10, 11]. However, the firms may suffer from its reputation if it fails to meet the promised delivery time. Thus, some firms propose compensation for the delayed delivery, such as offering vouchers and credits [12, 13]. Therefore, delivery time needs to be carefully addressed for firms to be competitive.

An important factor for a firm to guarantee a reliable delivery time to consumers lies in the availability of products that the firm needs to complete customer orders in a timely manner. Maintaining inventory levels to ensure that products can be sent to customers is an effective way in fulfilling the availability of products [3]. Otherwise, the firm could make a poor impression on some customers and even damage its credibility due to delayed delivery induced by stockout. Inventory control policies have been investigated based on the trade-off between set-up costs and holding costs. However, with the delivery time as an important competitive advantage for firms to attract customers, the following reasons should be included into the inventory control policies in order to provide a better service [14]. On one hand, keeping sufficient products in inventory enables a quick delivery of the products to customers, and the quick delivery enhance customer satisfaction and loyalty, especially for impatient customers. On the other hand, in an oligopoly market where customers are sensitive to delivery speed, holding inventory allows firms to deliver their product earlier and to avoid damaging their competitive position. Besides the beneficial effects above for holding inventory, stocking products excessively incurs additional costs. Therefore, how to integrate inventory decisions with delivery time is an important problem in a competitive oligopolistic market.

In addition to such inter-firm competition, intra-firm conflict will also affect operation of firms. The intra-firm conflict represents the actions of one functional department frustrate another functional department [15]. For instance, the marketing and operations departments represent the key functional areas of a modern firm, and impact on producing and delivering products to customers [16]. The marketing department is regarded as a revenue centre that controls marketing factors, while the operations department is regarded as a cost centre that aims to reduce costs and operational efficiency [17, 18]. The conflict between the two functional departments can be commonly seen from a mismatched forecast of demand for the products, where the marketing department tends to assume a quick raise or drop of production according to the market while the actual production managed by the operations department may be inflexible instead. Meanwhile, the collaborative operation of the functional departments determine the inventory and consequently the delivery time to cunstomers. Therefore, how to deal with the intra-firm conflict, i.e. marketing and operations departments, is an important problem faced by firms in competitive environment.

Our investigation emphases on the development of time-inventory competition in an asymmetric oligopolistic market where every firm offers different delivery time and the corresponding order quantity to compete for time-sensitive customers. The behaviors of the firms are based on both intra-firm and inter-firm competition. From the intra-firm competition perspective, the marketing department for a given firm determines the delivery time as well as additional decision variable price to capture the total demand by increasing the cost investment, while the operations department complies the aim of the marketing department by controlling the whole inventory cost. From the perspective of inter-firm competition in the oligopoly market, the marketing department of each firm aims to shorten delivery time to compete for time-sensitive customers, which is described in this paper by a market share attraction model relative to delivery time. According to the demand forecast, the operations department of each firm uses the economic order quantity model to guarantee on-time delivery. In view of the above observations, we focus on the following research questions: (i) What are the optimal intra-firm strategy of a given firm that adopts the decentralization and centralization of marketing and operations departments with respect to make delivery time and order quantity, respectively? (ii) How will a firm adjust its equilibrium delivery time and order quantity when marketing or operations factors change in oligopoly market? (iii) How does a dominant firm adopt the delivery time policy in a cooperative oligopoly market? (iv) What is the impact of different marketing or operations factors on equilibrium price, delivery time, and order quantity in an oligopoly market when customers are sensitive to both delivery time and price?

The primary contributions of this paper are as follows. First, to the best of our knowledge, this is the first to jointly study time and inventory: (i) in decentralized intra-firm scenario where the marketing department maximize revenue while the operations department minimize the total inventory cost; (ii) in a centralized intra-firm scenario where the marketing and operations departments joint maximize the total profit. By comparison, results suggest that the optimal intra-firm strategy is in the centralized scenario regardless of the longer delivery time than that in the decentralized scenario. Second, we utilize a supermodular games approach to analyze the unique Nash equilibrium derived from an oligopolistic competitive inventory model with time-dependent demand. This paper presents the directional impact of changes in marketing and operations factors on delivery time and ordered quantity in a non-dominated oligopoly market (every firm market share <50%) and in a dominated oligopoly market (one firm market share ≥ 50%), respectively. The results can be extended to the discussion of the incorporated additional decision price. Third, we explore the delivery time strategy of a leader firm under the cooperative competition with and without asymmetry. The analysis show that it is beneficial for the firms with high competitive strength to adopt the delivery time strategy in asymmetric oligopolistic market, but it fails to do so for the firm with the low competitive strength. On the other hand, no firm benefits from the delivery time strategy in symmetric oligopolistic market. Finally, numerical experiments are performed to estimate the effect of changes in marketing and operations factors on the optimal results, such as delivery time, order quantity, price, market share, and profit. We find that the marketing factors play a more important role in affecting equilibrium measures than operations factors.

The remainder of this paper is organized as follows. The next section offers a brief review of the delivery time literature. Section 3 describes a time and inventory competition model and presents a self-sensitivity analysis by outlining the theory underlying the supermodular game approach and a cross-sensitivity analysis in a symmetric duopoly that adopts a traditional approach. Section 4 models a mode of cooperative competition. Section 5 describes the extension of our basic model to incorporate price as an additional decision variable. The aim of Section 6 is to identify the effects of changes in marketing and operations factors on delivery time, price and order quantity decision variables through numerical experiments. Management insights are discussed in Section 7. The final section provides a brief conclusion and suggests directions for future research. All proofs are found in the Appendix.

## Literature review

This study is related to three streams of literature. The first stream examines the importance of delivery time performance under different competition modes, the second stream examines delivery time in inventory management, and the final stream examines the marketing and operations interface. We summarize these literature in Table 1.

**Table 1:** Related literature.

Research papers	Decision variables			market structures			Marketing-operations interface
	Delivery time	Price	Inventory	Monopoly	Duopoly	Oligopoly	
So and Song (1998) [1]	✓	✓		✓			
Ray and Jewkes (2004) [19]	✓			✓			
Li et al. (2014) [20]	✓	✓		✓			
Qian (2014) [6]	✓	✓		✓			
Boyaci and Ray (2003) [5]	✓	✓		✓			
Nguyen and Wright (2015) [2]	✓			✓			
Lederer and Li (1997) [21]	✓	✓				✓	
So (2000) [22]	✓	✓				✓	
Bernstein and Federgruen (2004) [23]	✓	✓				✓	
Shang and Liu (2011) [24]	✓					✓	
Li(1992) [14]	✓		✓			✓	
Hill and Khosla(1992) [25]	✓		✓	✓			
Ching(2001) [12]	✓		✓	✓			
Yang and Pan (2004) [26]	✓		✓	✓			
Yang and Geunes (2007) [27]	✓		✓	✓			
Selvarajah and Steiner (2009) [28]	✓	✓	✓	✓			
Jha, and Shanker(2013) [29]	✓		✓			✓	
Sadjadi et al. (2016) [30]	✓		✓			✓	
Yang et al.(2017) [31]	✓		✓		✓		
Chatterjee et al. (2002) [38]	✓		✓	✓			✓
Ho and Zheng (2004) [39]	✓				✓		✓
Pekgün et al. (2008) [40]	✓	✓		✓			✓
Our paper	✓	✓	✓			✓	✓

The first stream is related to the literature on the importance of delivery time performance under different competition modes. There are two categories of competitions among the relevant studies that have discussed delivery time. First, there is a rich body of work delivery time in a monopolistic competition. So and Song (1998) [1] developed a mathematical framework to determine the joint optimal price, delivery time guarantee and capacity expansion decision variables in a service industry, and found that the industry selects the best competing strategies under different operating characteristics. Different from the work of So and Song (1998) [1], Ray and Jewkes (2004) [19] considered price as a linear function of the length of the delivery time, and indicate that ignoring the relation between price and delivery time will generate suboptimal decisions. Li et al. (2014) [20] studied how a risk-averse firm determine price and promised delivery time in two scenarios, including one scenario for independent price and promised delivery time and the other scenario for promised delivery time sensitive price. Qian (2014) [6] extended the above literature to assume the demand to be dependent ont only on price and delivery time but also on service level and other quality-like performance, and found that better profitability can be achieved by coordinating operations and marketing factors. Boyaci and Ray (2003) [5] investigated a firm selling two substitutable products that are different from their prices and delivery times. They studied the effect of capacity-related costs on optimal delivery times and price decisions in different scenarios where the firm is constrained in capacity for none, one, or both product(s). Different from previous research, Nguyen and Wright (2015) [2] established a profit-maximizing mathematical model to determine an optimal quoted delivery time and capacity level by taking the time variation of demand into account. Second, some literature focused on the importance of delivery time in a oligopolistic competition. Lederer and Li (1997) [21] studied competition between *n* firms that produce goods for customers sensitive to price and delivery time. They proved the competitive equilibrium is well defined under two special case: one case where firm are differentiated by cost, mean processing time and processing time variability, and the other case where firm are differentiated by cost and mean processing time but customers are differentiated by demand function and delay sensitivity. So (2000) [22] developed a stylized model to analyze the impact of using delivery time guarantees in a multi-firm competition, and suggested that the equilibrium price and time guarantee decisions in an symmetric oligopolistic market behave in a similar fashion as the optimal solution in a monopolistic situation, while these firms will exploit their distinctive firm characteristics to compete for customers sensitive price and delivery time in heterogeneous market. Bernstein and Federgruen (2004) [23] developed a stochastic general equilibrium inventory model for an oligopoly where distributions are functions of all retailers’ prices and all retailers’ service levels, and they proceeded with the investigation of the equilibrium behavior of infinite-horizon models for industries facing price competition only, simultaneous price and service-level competition and two-stage competition. Shang and Liu (2011) [24] investigated firms’ competitive behaviors in industries where customers are sensitive to both promised delivery time and quality of service measured by the on-time delivery rate. They construct an oligopoly game with an external quality of service under the competition in promised delivery time at the marketing level, and a two-stage game under the competition in capacity at the strategic level. Although the available literature have investigated the decisions of delivery time and price under monopolistic or oligopolistic competitive market, there is a lack of investigation on the interaction between delivery time and inventory that is explicitly handled in our study.

The second stream involves delivery time in inventory management. Li(1992) [14] explored the role of inventory in delivery time competition. Hill and Khosla(1992) [25] discussed the costs and benefits of lead time reduction in an inventory and production planning model. Ching(2001) [12] used optimal (*s*, *S*) policy for production planning with delivery time guarantees in one-machine manufacturing system and derived an analytical form of the steady-state probability distribution of the inventory level. Yang and Pan (2004) [26] presented an integrated inventory model with controllable lead time, and found that lead time can be shorter. Yang and Geunes (2007) [27] studied a continuous review inventory replenishment system to deal with how a supplier determine order lead time and price of a product. Further, some researchers have investigated supply chain consisting of a supplier(s) and a retailer(s) addressing delivery time and inventory management problems. Selvarajah and Steiner (2009) [28] used an approximation algorithms for the supplier’s supply chain scheduling problem to minimize delivery and inventory holding costs. Jha, and Shanker(2013) [29] formulated a production-inventory model with controlled lead time and service level constraints to coordinate a two-echelon system with single vendor and multiple buyers. Sadjadi et al. (2016) [30] applied queuing approach to study a stochastic location-inventory problem with the consideration of delivery time. Yang et al.(2017) [31] extended the Newsvendor model to study inventory competition in a dual-channel supply chain with stockout-out-based consumer switching behavior and with consideration of delivery time in online channel. Compared to a variety of studies that considered the delivery time and inventory have been performed under a monopoly market, research for a more practical oligopolistic competitive market is scarce. By considering the delivery time and inventory under oligopoly competition, this investigation would enrich the literature and offer more insight into the firm decisions.

The final stream involves marketing and operations interface literature. Because of labour specializations, the objective of the marketing department is to create demand, while the goal of the operations department is to match and fulfill demand Tang(1990) [32]. On the one hand, there is a large body of literature on the conflict between these functional areas. Piercy(2007) [33] presented six key issues that result in poor marketing-operations relationships. Later, Piercy(2010) [34] proposed three approaches address these poor relationships. On the other hand, some papers develop a theoretical framework. Erickson(2011) [35] explored a differential game model for the marketing-operations interface. Liu et al.(2014) [36] focused on the impact of transfer pricing on the decision variables in marketing and operations departments. Sale et al. (2017) [37] used a dynamic marketing-operations interface to examine the effects of variations in marketing and operations parameters on both optimal profits and optimal product lifestyle length. Recent years have seen growing attention paid to the study of marketing and operations interface literature on delivery time. Chatterjee et al. (2002) [38] modelled the decision-making process of a firm that the marketing department makes delivery commitments to the customers and the operations department produces and deliver the product. Ho and Zheng (2004) [39] integrated the gap model of service quality from marketing with the classical queueing models from operations, and derived the existence of Nash equilibria in a duopolistic game and showed that the delivery-time game is similar to a Prisoners’ Dilemma when the cost of adding capacity is small Pekgün et al. (2008) [40] studied how to coordinate the marketing and operations departments where the former decides price and the latter sets delivery time. With the interactions between the marketing and operations departments increasingly incorporated in literature to estimate the optimal delivery time and/or inventory under either monopolistic or duopolistic market, this paper is the first to investigate the competition under an oligopolistic competitive market with the time-sensitive customers.

## Model development and analysis

There are n firms competing in a market where customers make the decision to purchase the products among the firms depending on one or more of the following factors, such as the delivery time, price, advertising effort, firm reputation, and so on. Hence, the conventional low price strategy does not necessarily guarantee a firm to excel in the market. Meanwhile, delivery time for the products is increasingly considered one of the most influential elements that affect the customers’ purchase behavior, and more and more competing firms begin to choose and advertise the delivery time for their products to customers. Thus, we in this paper explicitly model the effect of delivery time on demand while other factors are fixed and do not affect the total demand. In Section 5, we will explore a general situation where delivery time and price are simultaneously regarded as the decision variables. Similar to previous studies, we assume that the inventory replenishment way of each firm follows the well-known economic order quantity (EOQ) model to meet the needs of customers in a timely fashion. Although with some restrictive assumptions (e.g., fixed costs and constant demand rate), EOQ model offers a robust approach that is able to manage parameter variability as shown in Wilson (1934) [41]. Furthermore, the study assumes that the marketing department announces the delivery time to customers and can obtain parameters information about the relative marketing factors, and the operations department determine the order quantity and obtain parameters information about the relative inventory factors. Each firm aims to maximize its profit in a non-cooperative oligopolistic market. The notations for the model is summarized in Table 2.

**Table 2:** Notation.

*p*_*j*_ = Selling price per unit of firm *j*.
*MC*_*j*_ = Marginal cost per unit of firm *j*.
*γ*_*j*_ = *p*_*j*_ − *mc*_*j*_, profit margin per unit sold of firm *j*.
*Q*_*j*_ = The order quantity each time of firm *j*.
*C*(*t*_*j*_) = Delivery time reduction cost function of firm *j*.
*C*_*oj*_ = Ordering cost per order of firm *j*.
*C*_*hj*_ = Inventory holding cost per unit held per unit time of firm *j*.
*F*_*j*_ = Fixed costs of firm *j*.
*m* = The overall market size.

**Fig 1:**
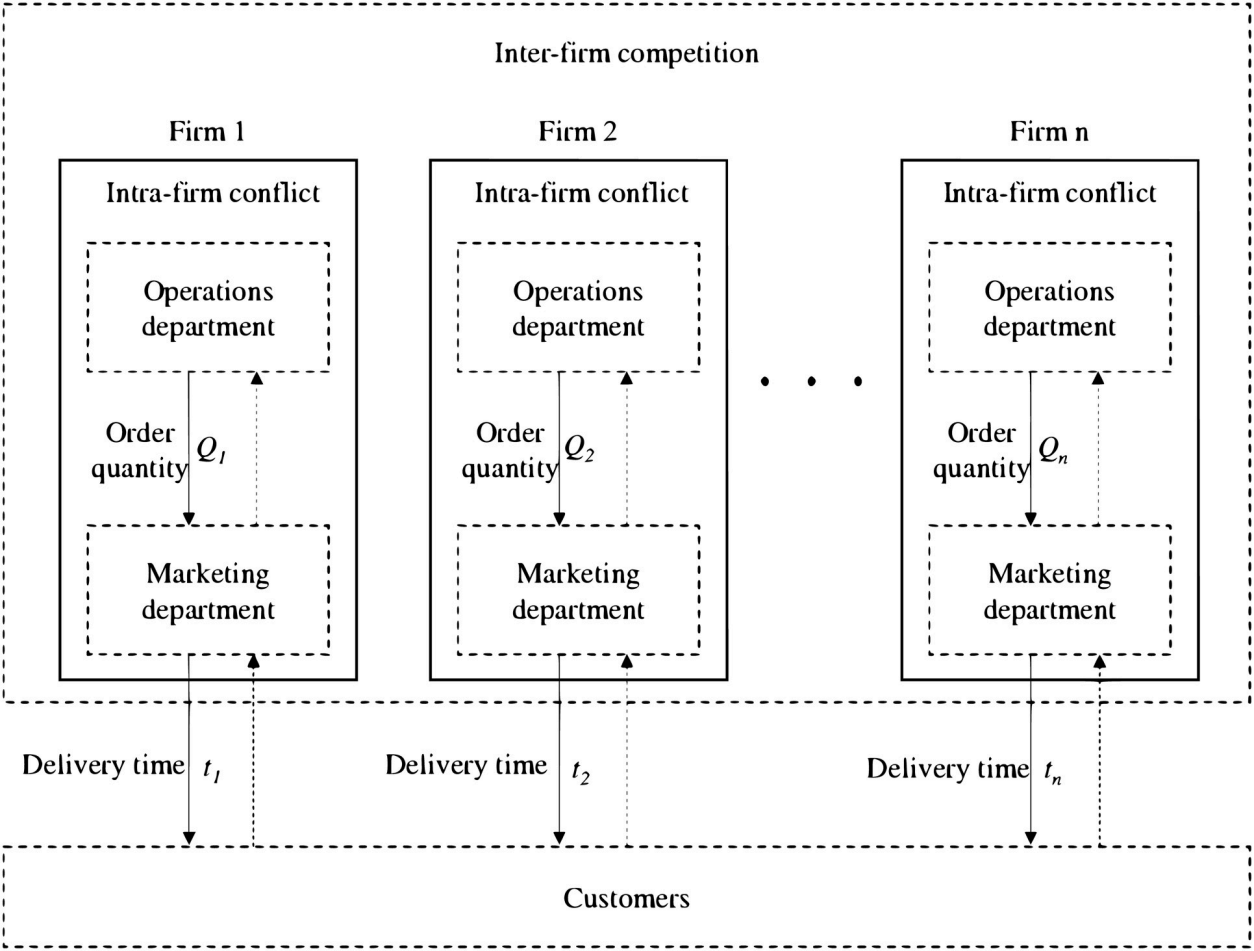
The structure of the proposed model.

The structure of the proposed model is presented in Fig 1. We use a market share attraction model to study competition on delivery time in an oligopoly market, where the overall market size is given as a fixed value of *m*. Under such a market setting, the decision of delivery time affects the market share of each firm but not the overall market size. The multiplicative competitive interaction (MCI) model is used to represent the market share of each firm as follows:

$$M_{j}=\frac{f_{j}\left(t_{j}\right)}{\sum_{i=1}^{n}f_{i}\left(t_{i}\right)}=\frac{\beta_{j}t_{j}^{-b_{j}}}{\sum_{i=1}^{n}\beta_{i}t_{i}^{-b_{i}}},$$ (1)

Here, the term $\beta_{j}t_{j}^{-b_{j}}$ represents the attraction of firm *j*, and the market share of each firm is given by the ratio of its attraction to the total attraction of all firms. The attraction of firm *j* reflects customers’ degree of satisfaction on the purchases over its delivery time and others factors, such as locations and goodwill. The parameter *b*_*j*_ represents a measure of the time attraction factor of firm *j*, and the larger value of *b*_*j*_ reflects a higher sensitivity of customers to delivery time. The parameter *β*_*j*_ refers to the aggregate effects of other factors excluding delivery time. Relative discussions about the market share models could be referred to Cooper and Nakanishi (1988) [42] or Lilien et al. (1992) [43].

In view of the above definitions, the profit function *π*_*j*_ of firm *j* per unit time is given by

$$\pi_{j}=\gamma_{j}\frac{mf_{j}\left(t_{j}\right)}{\sum_{i=1}^{n}f_{i}\left(t_{i}\right)}-C\left(t_{j}\right)-(\frac{C_{oj}}{Q_{j}}\frac{mf_{j}\left(t_{j}\right)}{\sum_{i=1}^{n}f_{i}\left(t_{i}\right)}+\frac{Q_{j}}{2}C_{hj})-F_{j},\;\;\;\;j=1,2,...,n.$$ (2)

The profit of firm *j* consists of four terms. The first term represents the firm’s marginal profit. The second term represents delivery time cost $C\left(t_{j}\right)=\delta_{j}t_{j}^{-\epsilon_{j}}$ that is assumed as a Cobb-Douglas production function [19, 25], where *δ*_*j*_ and *ϵ*_*j*_ are positive constants with *δ*_*j*_ > 0 and *ϵ*_*j*_ ≥ 0. According to Eq (2), the traditional total inventory cost per unit *TC*_*j*_ is the sum of ordering and holding costs, which is the third term as shown in bracket. The last term is the fixed cost, such as facilities cost, set-up cost and advertise cost, which is independent on the delivery time. Hence, ignoring the term does not affect the following results. In this paper, in addition to the above oligopoly competition, we discuss the intra-firm conflict for each firm. The operations department of each firm determines order quantity and aims to minimize the total inventory costs, namely

$$TC_{j}=\frac{C_{oj}}{Q_{j}}\frac{m\beta_{j}t_{j}^{-b_{j}}}{\sum_{i=1}^{n}\beta_{i}t_{i}^{-b_{i}}}+\frac{Q_{j}}{2}C_{hj},\;\;\;\;j=1,2,...,n.$$ (3)

Whereas the marketing department determines delivery time and aims at maximizing the sum of the first and second terms in Eq 2, namely

$$\pi_{j}^{r}=\gamma_{j}\frac{m\beta_{j}t_{j}^{-b_{j}}}{\sum_{i=1}^{n}\beta_{i}t_{i}^{-b_{i}}}-\delta_{j}t_{j}^{-\epsilon_{j}}-F_{j},\;\;\;\;j=1,2,...,n.$$ (4)

Meanwhile, all model parameters are classified into two groups: marketing factors that are affected by the marketing department, including *γ*_*j*_, *β*_*j*_, *b*_*j*_, *δ*_*j*_ and *ϵ*_*j*_, and operations factors that are affected by the operations department, including *C*_*hj*_ and *C*_*oj*_. In the centralized scenario, the marketing department and the operations department cooperate and jointly maximize Eq 2. By contrast, in the decentralized scenario, the marketing and operations departments separately maximize their own profits and minimize their own costs using Eqs 3 and 4, respectively. Let us first compare the two scenarios by means of the Nash equilibrium solution of game theory such that no single firm can benefit by unilaterally deviating from this equilibrium point.

**Proposition 1** *In an asymmetric oligopoly, the following results hold*:

(i) *The equilibrium delivery time of each firm under centralization is longer than that under decentralization*.
(ii) *The equilibrium order quantity of each firm, under centralization or decentralization*, *lies on an n-dimensional ellipsoid* $\frac{2mC_{hj}}{C_{oj}}\sum_{j=1}^{n}Q_{j}^{2}=1.$
(iii) *The equilibrium total inventory cost TC*_*j*_ *of each firm*, *under centralization or decentralization*, *lies on an n-dimensional ellipsoid* $\frac{\sum_{j=1}^{n}TC_{j}^{2}}{2mC_{oj}C_{hj}}=1.$
(iv) *The optimal profit is higher under centralization than under decentralization*.

Result (i) suggests that there exists a necessary condition for an interior solution at optimality (∂*π*_*j*_/∂*t*_*j*_ = 0) under the centralization between marketing and operations departments. However, there is no necessary condition under decentralization. Hence, the firm would offer a longer optimal delivery time under centralization than that under decentralization. Result (ii) states that there exists at least one firm under both coordination and decentralization that has a higher equilibrium order quantity. The reasoning for result (iii) is analogous to that for result (ii). Result (iv) suggests that each firm prefers to coordinate its effort to serve its overall interest than to have the marketing and operations departments operate in a decentralized fashion. It is intuitive that the centralized scenario can achieve the information sharing between the marketing and operations departments and then better coordinate the intra-firm conflict. Hence, it is reasonable to believe that the firm can implement a longer delivery time to get the higher profit under centralization than under decentralization, which is different from Pekgün et al. (2008) [40] where the inefficiencies created by decentralization are attributed to a longer delivery time and lower profit than in the centralized setting. As a result, our findings provide new insights for firms regarding the interaction of market and operations department. In what follows, we primarily analyze the optimal results under the centralized scenario.

The following two propositions provide further results regarding how changes in the various factors, namely, the marketing and operations factors, for a given firm *j* affect the behaviours of its equilibrium centralized delivery time $t_{j}^{**}$ and order quantity $Q_{j}^{**}$ in non-dominated and dominated asymmetric oligopolies. In the proof of Proposition 1, we do not obtain the analytical solution of $t_{j}^{**}$ and $Q_{j}^{**}$. As a consequence, we introduce the following supermodular game approach to verify a related proposition, which is a popular approach in the economics literature to address comparative statics in game theory problems [44].

**Lemma 1** *In a smooth supermodular game with n firms*, *the following assumptions hold for each firm i and each rival j*.

(i) *The profit equation is twice continuously differentiable with respect to t*_*j*_ *and Q*_*j*_.
(ii) *Complementary strategies* (*of each firm’s own strategic variables*): $\frac{\partial^{2}\pi_{j}}{\partial t_{j}\partial Q_{j}}\geq 0$.
(iii) *Strategic complements* (*for strategic variables between firms*): $\frac{\partial^{2}\pi_{j}}{\partial t_{j}\partial t_{i}}\geq 0,\frac{\partial^{2}\pi_{j}}{\partial t_{j}\partial Q_{i}}\geq 0,\frac{\partial^{2}\pi_{j}}{\partial Q_{j}\partial t_{i}}\geq 0,\frac{\partial^{2}\pi_{j}}{\partial Q_{j}\partial Q_{i}}\geq 0.$
(iv) *Complementary policy parameter* $\theta :\frac{\partial^{2}\pi_{j}}{\partial t_{j}\partial \theta }\geq 0$ *and* $\frac{\partial^{2}\pi_{j}}{\partial Q_{j}\partial \theta }\geq 0.$
  *Under these conditions and assuming a unique Nash equilibrium*, *the following comparative static results hold for all firms*
  $$\frac{\partial t_{j}^{**}}{\partial \theta }\geq 0;\frac{\partial Q_{j}^{**}}{\partial \theta }\geq 0.$$

Now, the results obtained are subjected to a supermodular games approach, and summarized in Propositions 2 and 3.

**Proposition 2** *In a centralized non-dominated asymmetric oligopoly*, *the equilibrium delivery time* $t_{j}^{**}$ *and the equilibrium order quantity* $Q_{j}^{**}$ *have the following properties*:

(i) $t_{j}^{**}$ *is decreasing and* $Q_{j}^{**}$ *is increasing with* *γ*_*j*_ *and β*_*j*_. *Furthermore*, $t_{j}^{**-b_{j}}$ *and* $Q_{j}^{**}$ *increase in b*_*j*_.
(ii) $t_{j}^{**}$ *is increasing and* $Q_{j}^{**}$ *is decreasing with δ*_*j*_ *and ϵ*_*j*_.
(iii) $t_{j}^{**}$ *is increasing and* $Q_{j}^{**}$ *is decreasing with C*_*hj*_.
(iv) $t_{j}^{**}$ *is decreasing and* $Q_{j}^{**}$ *is increasing with m*.

Result (i) asserts that increased marginal profit will encourage a firm to offer a shorter delivery time, and then the demand of the firm will increase. Therefore, the firm should order more commodities to satisfy the total demand of customers. As the aggregate effects of other factors increase, such as more convenient service locations, the optimal response of the firm is to provide a shorter delivery time. However, the theoretical analysis does not directly show the impact of the time attraction factor on delivery time. This gap can be filled using numerical experiments. Result (ii) indicates that if the parameters associated with delivery time reduction costs increase, then the optimal response for a firm is to prolong its delivery time and decrease its order quantity to mitigate the effect on its profit. Result (iii) implies that if the inventory holding cost of a firm increases, then it should increase its delivery time and decrease its order quantity as an optimal response to reduce inventory pressures. Finally, result (iv) confirms the intuitive result that as the size of the overall market increases, all firms will offer a shorter delivery time to compete for market share and raise their order quantity to satisfy the increased demand. Due to the intractable analysis, we will obtain the properties of the unit ordering cost of a firm by conducting the duopoly or a numerical simulation.

**Proposition 3** *For a centralized dominated asymmetric oligopoly*, *the equilibrium delivery time* $t_{j}^{**}$ *and order quantity* $Q_{j}^{**}$ *have the following properties*:

(i) $t_{j}^{**}$ *and* $Q_{j}^{**}$ *decrease in γ*_*j*_ *and b*_*j*_ *and increase in β*_*j*_.
(ii) $t_{j}^{**}$ *and* $Q_{j}^{**}$ *increase in δ*_*j*_ *and ϵ*_*j*_.
(iii) $t_{j}^{**}$ *and* $Q_{j}^{**}$ *decrease in C*_*hj*_ *but increase in C*_*oj*_.

The changes in the equilibrium delivery time and order quantity of a firm in a dominated oligopoly behave differently compared with those of a firm in a non-dominated oligopoly. Consequently, the firm should take different measures in response to changes in marketing and operations factors. So and Song (1998) [1], Ray and Jewkes (2004) [19] and Ho and Zheng (2004) [39] have discussed the impact of model parameters on the equilibrium delivery time. However, they ignore the impact of inventory factors on the delivery time, and this problem is addressed in our paper.

Despite that a supermodular game approach provides a theoretical method for obtaining sensitivity analysis of the optimal strategies, it only indicates the direction of changes reflected in the optimal results and not the magnitude or impact of the optimal results $t_{i}^{**}$ and $q_{i}^{**}$ (*i* ≠ *j*) for other firms (cross-sensitivity analysis). The above disadvantage could be overcome by the implicit function theorem. Unfortunately, when the number of decision variables or firms increase *n* ≥ 2, the theorem becomes rather difficult to apply because it suffers from the so-called curse of dimensionality. Consequently, we may consider the simplified case below of a cross-sensitivity analysis under symmetric duopoly.

In light of Eq (2), the profit function of firm *j* = 1, 2 per unit time is given by

$$\begin{array}{c} \begin{array}{cc} & \pi_{1}=\left(\gamma_{1}-\frac{C_{o1}}{Q_{1}}\right)\frac{m\beta_{1}t_{1}^{-b_{1}}}{\beta_{1}t_{1}^{-b_{1}}+\beta_{2}t_{2}^{-b_{2}}}-\delta_{1}t_{1}^{-\epsilon_{1}}-\frac{Q_{1}}{2}C_{h1}-F_{1},\\ & \pi_{2}=\left(\gamma_{2}-\frac{C_{o2}}{Q_{2}}\right)\frac{m\beta_{2}t_{2}^{-b_{2}}}{\beta_{1}t_{1}^{-b_{1}}+\beta_{2}t_{2}^{-b_{2}}}-\delta_{2}t_{2}^{-\epsilon_{2}}-\frac{Q_{2}}{2}C_{h2}-F_{2}. \end{array} \end{array}$$ (5)

It follows from Proposition 1 that there exists a unique Nash equilibrium in Eq (5). Therefore, the first-order optimality conditions for an optimal solution ($t_{j}^{**},Q_{j}^{**}>0$) satisfy the following:

$$\frac{\partial \pi_{j}}{\partial t_{j}}=0,\;and\;\;\frac{\partial \pi_{j}}{\partial Q_{j}}=0,\;\;\;j=1,2.$$ (6)

A theoretical derivation is described by the impact of the changes in each of the parameters *γ*_*j*_, *β*_*j*_, *b*_*j*_, *δ*_*j*_, *ϵ*_*j*_, *C*_*hj*_ and *C*_*oj*_ for a given firm *j* = 1, 2 on its equilibrium delivery time $t_{j}^{**}$ and order quantity $q_{j}^{**}$ as well as on the equilibrium $t_{i}^{**}$ and $q_{i}^{**}$ (*i* ≠ *j*) of its competitors. Let *θ* be one of the parameters *γ*_*j*_, *β*_*j*_, *b*_*j*_, *δ*_*j*_, *ϵ*_*j*_, *C*_*hj*_ or *C*_*oj*_. Both sides of Eq (6) are partially differentiated with respect to *θ*, as given by

$$\begin{array}{c} \begin{array}{cc} & \frac{\partial^{2}\pi_{1}}{\partial t_{1}^{2}}\frac{\partial t_{1}}{\partial \theta }+\frac{\partial^{2}\pi_{1}}{\partial t_{1}\partial Q_{1}}\frac{\partial Q_{1}}{\partial \theta }+\frac{\partial^{2}\pi_{1}}{\partial t_{1}\partial t_{2}}\frac{\partial t_{2}}{\partial \theta }+\frac{\partial^{2}\pi_{1}}{\partial t_{1}\partial Q_{2}}\frac{\partial Q_{2}}{\partial \theta }+\frac{\partial^{2}\pi_{1}}{\partial t_{1}\partial \theta }=0,\\ & \frac{\partial^{2}\pi_{1}}{\partial Q_{1}\partial t_{1}}\frac{\partial t_{1}}{\partial \theta }+\frac{\partial^{2}\pi_{1}}{\partial Q_{1}^{2}}\frac{\partial Q_{1}}{\partial \theta }+\frac{\partial^{2}\pi_{1}}{\partial Q_{1}\partial t_{2}}\frac{\partial t_{2}}{\partial \theta }+\frac{\partial^{2}\pi_{1}}{\partial Q_{1}\partial Q_{2}}\frac{\partial Q_{2}}{\partial \theta }+\frac{\partial^{2}\pi_{1}}{\partial Q_{1}\partial \theta }=0,\\ & \frac{\partial^{2}\pi_{2}}{\partial t_{2}\partial t_{1}}\frac{\partial t_{1}}{\partial \theta }+\frac{\partial^{2}\pi_{2}}{\partial t_{2}\partial Q_{1}}\frac{\partial Q_{1}}{\partial \theta }+\frac{\partial^{2}\pi_{2}}{\partial t_{2}^{2}}\frac{\partial t_{2}}{\partial \theta }+\frac{\partial^{2}\pi_{2}}{\partial t_{2}\partial Q_{2}}\frac{\partial Q_{2}}{\partial \theta }+\frac{\partial^{2}\pi_{2}}{\partial t_{2}\partial \theta }=0,\\ & \frac{\partial^{2}\pi_{2}}{\partial Q_{2}\partial t_{1}}\frac{\partial t_{1}}{\partial \theta }+\frac{\partial^{2}\pi_{2}}{\partial Q_{2}\partial Q_{1}}\frac{\partial Q_{1}}{\partial \theta }+\frac{\partial^{2}\pi_{2}}{\partial Q_{2}\partial t_{2}}\frac{\partial t_{2}}{\partial \theta }+\frac{\partial^{2}\pi_{2}}{\partial Q_{2}^{2}}\frac{\partial Q_{2}}{\partial \theta }+\frac{\partial^{2}\pi_{2}}{\partial Q_{2}\partial \theta }=0. \end{array} \end{array}$$ (7)

Note that all of the second partial derivatives of *π*_*j*_ with respect to the equilibrium $t_{j}^{**}$ and $q_{j}^{**}$ (*j* = 1, 2) as well as each parameter *θ* exist and are continuous. Furthermore, Eq (7) can be rewritten in matrix form as follows:

$$\begin{array}{c} (\begin{array}{c} \frac{\partial t_{1}}{\partial \theta }\\ \frac{\partial Q_{1}}{\partial \theta }\\ \frac{\partial t_{2}}{\partial \theta }\\ \frac{\partial Q_{2}}{\partial \theta } \end{array})=-H^{-1}(\begin{array}{c} \frac{\partial^{2}\pi_{1}}{\partial t_{1}\partial \theta }\\ \frac{\partial^{2}\pi_{1}}{\partial Q_{1}\partial \theta }\\ \frac{\partial^{2}\pi_{2}}{\partial t_{2}\partial \theta }\\ \frac{\partial^{2}\pi_{2}}{\partial Q_{2}\partial \theta } \end{array}), \end{array}$$ (8)

where

$$\begin{array}{c} H=(\begin{array}{cccc} \frac{\partial^{2}\pi_{1}}{\partial t_{1}^{2}} & \frac{\partial^{2}\pi_{1}}{\partial t_{1}\partial Q_{1}} & \frac{\partial^{2}\pi_{1}}{\partial t_{1}\partial t_{2}} & \frac{\partial^{2}\pi_{1}}{\partial t_{1}\partial Q_{2}}\\ \frac{\partial^{2}\pi_{1}}{\partial Q_{1}\partial t_{1}} & \frac{\partial^{2}\pi_{1}}{\partial Q_{1}^{2}} & \frac{\partial^{2}\pi_{1}}{\partial Q_{1}\partial t_{2}} & \frac{\partial^{2}\pi_{1}}{\partial Q_{1}\partial Q_{2}}\\ \frac{\partial^{2}\pi_{2}}{\partial t_{2}\partial t_{1}} & \frac{\partial^{2}\pi_{2}}{\partial t_{2}\partial Q_{1}} & \frac{\partial^{2}\pi_{2}}{\partial t_{2}^{2}} & \frac{\partial^{2}\pi_{2}}{\partial t_{2}\partial Q_{2}}\\ \frac{\partial^{2}\pi_{2}}{\partial Q_{2}\partial t_{1}} & \frac{\partial^{2}\pi_{2}}{\partial Q_{2}\partial Q_{1}} & \frac{\partial^{2}\pi_{2}}{\partial Q_{2}\partial t_{2}} & \frac{\partial^{2}\pi_{1}}{\partial Q_{2}^{2}} \end{array}). \end{array}$$

Matrix *H* is non-singular and all derivations associated with Eq (8) are shown in the Appendix. Furthermore, the necessity of the existence of a unique Nash equilibrium for Eq (6) is the negative semi-definiteness of matrix *H* asserted by Gruca et al. (1992) [45], which is verified in the Appendix. Before introducing the following proposition, we first define some terms below:

$$\begin{array}{c} X=\frac{mC_{o}}{Q^{2}}\frac{f^{\prime }}{4f}<0,\\ Y=m\left(\gamma -\frac{C_{o}}{Q}\right)\frac{f^{\prime \prime }f-f^{\prime 2}}{4f^{2}}-\delta \varepsilon \left(\varepsilon +1\right)t^{-\epsilon -2}<0,\\ \Delta_{1}=-\frac{YmC_{o}}{Q^{3}}-X^{2}>0. \end{array}$$

**Proposition 4** *In a symmetric duopoly, the equilibrium delivery time* $t_{j}^{**}$ *and order quantity* $Q_{j}^{**}$ *have the following properties*, *where j* = 1, 2 *and i* ≠ *j*:

(i) *When γ*_*j*_, *β*_*j*_ *or* *b*_*j*_ *increase*, $t_{j}^{**}$ *decreases and* $Q_{j}^{**}$ *increases*, *but* $t_{i}^{**}$ *increases and* $Q_{i}^{**}$ *decreases*.
(ii) *When δ*_*j*_ *or ϵ*_*j*_ *increases*, $t_{j}^{**}$ *increases and* $Q_{j}^{**}$ *decreases*, *but* $t_{i}^{**}$ *decreases and* $Q_{i}^{**}$ *increases*.
(iii) *When C*_*hj*_ *increases*, $t_{j}^{**}$ *increases and* $Q_{j}^{**}$ *decreases*, *but* $t_{i}^{**}$ *decreases and* $Q_{i}^{**}$ *increases*.
(iv) *When C*_*oj*_ *increases*, $t_{j}^{**}$ *increases and* $Q_{j}^{**}$ *increases if* Δ_1_ − 2*X*^2^ < 0 *and* $Q_{j}^{**}$ *decreases if* Δ_1_ − 2*X*^2^ > 0, *but* $t_{i}^{**}$ *decreases and* $Q_{i}^{**}$ *increases*.

The findings show that the signs of the self-sensitivity analysis are the same as those in Proposition 2. Furthermore, the optimal response of one firm is always the opposite of that of the other firms. For example, a firm should shorten the delivery time and increase the order quantity to improve its profit, whereas the optimal response of its competitor is to take the opposite course to improve its profit when the related parameters of result (i) increase. If the cost factors related to shortening the delivery time increase, then the optimal response for the firm is to prolong the guaranteed delivery time and decrease the order quantity to temper the pressure on its time cost, whereas its competitor should seize this opportunity to shorten its delivery time and increase its order quantity, which leads to more profits (result (ii)). Similar responses are presented in result (iii). However, if the per order ordering cost of a firm increases, then its optimal response should favour prolonging the delivery time. The optimal response for the order quantity varies across different parameter regions. The competitor will regard this situation as an opportunity to shorten its delivery time and increase its order quantity, which is shown in result (iv). In comparison to the study by Ha et al. (2003) [46] that investigates only the impact of operations factors (*C*_*h*_, *C*_*o*_) on the optimal delivery time, the present study attains additional insights into the decision of optimal delivery time by considering the changes in the marketing and operations factors. Moreover, it is found that the optimal order quantity would vary in response to changes in the marketing factors.

## Cooperative competition

In previous sections, non-cooperative oligopolistic competition based on delivery time and inventory are discussed. In this section, we examine how the asymmetric firms determine the delivery time strategy when the competition changes from non-cooperation to cooperation. This situation is similar to a firm leading the competition through merger and acquisition [47]. Thus, under cooperative competition, the leader firm determines $t_{j}^{***}\geq 0$ and $Q_{j}^{***}\geq 0$, *j* = 1, 2, 3…*n* and maximizes the joint profit *π*_*cc*_ by

$$\pi_{cc}=\sum_{j=1}^{n}\pi_{jcor},$$

where *π*_*jcor*_ is the centralized profit of firm *j* as expressed by (2). Therefore, we can obtain the following two propositions.

**Proposition 5** *Under cooperative competition among n firms*, *the optimal delivery time* $t_{j}^{***}$ *and order quantity* $Q_{j}^{***}$ *have the following properties*:

(i) $t_{j}^{***}\geq t_{j}^{**}$,
(ii) $Q_{j}^{***}$ *and* $Q_{j}^{**}$ *lie on the surface of n-dimensional ellipsoid* $\frac{C_{h}}{2mC_{o}}\sum_{j=1}^{n}Q_{j}^{2}=1,$ *and*
(iii) $\pi_{cc}^{***}>\pi_{cor}^{**}=\sum_{j=1}^{n}\pi_{jcor}^{**}.$

Proposition 5 asserts that the cooperative competition is beneficial to all firms, as the total profit is greater primarily due to the reduced cost by all firms with longer delivery time than that under non-cooperative centralized competition. The results of Proposition 5 are in consistence with the results from Mukherjee et al. [48], where it is reported that operation coordination are achieved through mergers and acquisitions with the benefits of improved productivity and reduced cost. In the following, Proposition 6 reveals the role of the delivery times of different firms when shifting from non-cooperative competition to cooperative competition. Let us introduce the following terms:

(i) Firm *j*′*s* total inventory cost per unit sold $\nu_{j}=\sqrt{\frac{2C_{oj}C_{hj}}{mM_{j}}},$
(ii) Firm *j*′*s* competitive strength $CS_{j}=\gamma_{j}-\frac{1}{2}\nu_{j},$
(iii) Weighted mean strength $WMS=\sum_{j=1}^{n}M_{j}\left(\gamma_{j}-\frac{1}{2}\nu_{j}\right).$

**Proposition 6** *Considering cooperative competition among n firms*,

(i) *With asymmetric firms*, *firm j will adopt a delivery time strategy if CS*_*j*_ > *WMS*, *otherwise*, *it will not*.
(ii) *With symmetric firms*, *none of the firms will adopt a delivery time strategy*.

Result (i) clarifies that when the competitive strength of a firm exceeds its weighted mean strength, it will adopt a suitable delivery time strategy. The reason is that this firm has competitive strength, which allows it to obtain more profit in a cooperative competition market, while other firms can free-ride, and thus, they will not adopt a delivery time strategy as subsidiaries. When the entire market share is equal to 1/n, there are no firms adopting a delivery time strategy to enhance their own profit because all market information is completely symmetric. Shortening a firm’s delivery time does not affect its market share, as result (ii) illustrates. Although the literature discuss the role of delivery time in non-cooperative oligopolistic market [13, 22–24], there is a lack of investigation of the cooperative competition in an oligopoly market. Investigation in this paper would help to fill this gap. From Propositions 5 and 6, we summarize the advantage of cooperative competition and how to adopt the optimal delivery time strategy under asymmetric and symmetric competitions.

## Extension: Introduction of price competition

In this section, we relax the assumption in the basic model by considering the exogenousity of price. In practice, price and delivery time have close relation, where the prices of the same product change with different delivery times. Take the United States Postal as an example, the express mail option delivers the product within 3-5 days at $23.95 and the priority mail option delivers the product within 6-9 days at $9.95. Based on the model of So (2000) [22], the attraction function of the firm *j* is now denoted as $g_{j}=\beta_{j}P_{j}^{-a_{j}}t_{j}^{-b_{j}}$, with *a*_*j*_, *b*_*j*_ ≥ 0 [22], and the parameter *a*_*j*_ is the price attraction factor of firm *j*. Thus, the profit of firm *j* takes the following form:

$$\pi_{j}=\left(P_{j}-MC_{j}-\frac{C_{oj}}{Q_{j}}\right)\frac{mg_{j}}{\sum_{i=1}^{n}g_{i}}-\delta_{j}t_{j}^{-\epsilon_{j}}-\frac{Q_{j}}{2}C_{hj}-F_{j},\;\;\;\;j=1,2,...,n.$$ (9)

Here, the marketing department will control both delivery time and price, whereas the operations department controls order quantity. Next, we will use the supermodular game method to conduct the self-sensitivity analysis under non-dominated and dominated oligopolies. However, Proposition 7 is confined to symmetric competition because the sensitivity analysis related to a non-dominated asymmetric oligopoly are difficult to obtain.

**Proposition 7** *For a non-dominated symmetric oligopoly*, the optimal delivery time t**, the optimal price P** and the optimal order quantity Q** *have the following properties*:

(i) t** *increases in a*, *δ* *and ϵ* *but decreases in MC*, *b*, *C*_*o*_ *or C*_*h*_.
(ii) P** *increases in MC*, *C*_*o*_ *or C*_*h*_ *but decreases in a*.
(iii) Q** *increases in C*_*o*_ *but decreases in C*_*h*_.

The impacts of the marketing and operations factors on the behaviour of the equilibrium delivery time, price and order quantity given in Proposition 7 are rather intuitive. A higher firm marginal cost will result in a higher price and a shorter delivery time. Moreover, as the price attraction factor increases, firms compete more intensely on price and therefore devote less effort to the delivery time, resulting in a longer delivery time. However, as the time attraction factor increases, firms are sufficiently willing to compete for time-sensitive customers to enhance their profits. For the parameters associated with the cost of the delivery time, the result is consistent with result (ii) of Proposition 2. Hence, there is a similar managerial implication. Finally, the increase of the operations factors leads to the decrease of equilibrium delivery time and the increase of the equilibrium price as well as the different influences on equilibrium order quantity.

**Proposition 8** *For a dominated asymmetric oligopoly*, *the equilibrium delivery time* $t_{j}^{**}$, *the equilibrium price* $P_{j}^{**}$ *and the equilibrium order quantity* $Q_{j}^{**}$ *have the following properties*:

(i) $t_{j}^{**}$, $P_{j}^{**}$ *and* $Q_{j}^{**}$ *increase in MC*_*j*_, *β*_*j*_, *δ*_*j*_, *ϵ*_*j*_ *and C*_*oj*_.
(ii) $t_{j}^{**}$, $P_{j}^{**}$ *and* $Q_{j}^{**}$ *decrease in a*_*j*_, *b*_*j*_ *and C*_*hj*_.

Proposition 8 is generalized to a dominated asymmetric oligopoly market by incorporating additional price variable are incorporated into the dominated asymmetric oligopolistic model. On one hand, we observe that the above results on the sensitivity analysis of the equilibrium delivery time and order quantity are in line with Proposition 3 which does not consider the price decision variable. Thus, the management insights are similar to Proposition 3 except for the equilibrium price. However, we find that the impact of the marketing and operations factors on the equilibrium price is consistent of the equilibrium delivery time and order quantity. It is intuitive that the optimal response of the dominated firm which has power to lead the market will increase the equilibrium price to earn more profit. Similarly, So (2000) [22] have studied how to determine price and delivery time for multi-firm to compete for market share in oligopoly market using the multiplicative competitive interaction model. In comparison, the present research not only considers a model for delivery time and price competition but also incorporates inventory competition in both symmetric and asymmetric oligopolies. Meanwhile, we use supermodular game to complete the sensitivity analysis of the equilibria in a dominated oligopoly instead of using the numerical simulation to provide the theoretic analysis. Moreover, compared with So (2000) [22], our paper extend to a general situation where each firm in the oligopolistic market implements different times and prices attraction factors, which attains some additional new management insights.

On the other hand, changes in dominated asymmetric firm parameters should elicit a response inconsistent with the findings of Proposition 7 where the characteristics of changes in the equilibrium are considered as a symmetric case. To verify the non-dominated asymmetric oligopoly case, there is a plausible situation in which inventory costs can be ignored, such as for items requiring little shelf space. Therefore, the impacts of changes in various parameters on the equilibrium are summarized below.

**Proposition 9** *For a non-dominated asymmetric service oligopoly*, *the equilibrium delivery time* $t_{j}^{**}$ *and the equilibrium order quantity* $P_{j}^{**}$ *have the following properties*:

(i) $t_{j}^{**}$ *decreases and* $P_{j}^{**}$ *increases with β*_*j*_ *and b*_*j*_.
(ii) $t_{j}^{**}$ *increases and* $P_{j}^{**}$ *decreases with a*_*j*_, *δ*_*j*_ *and ϵ*_*j*_.

Result (i) demonstrates that as the aggregate effects of other factors increase, such as a closer service location, a firm can shorten the delivery time but needs to charge a higher price to compensate for the cost of the shortened delivery time. However, as the time attraction factor increases, a firm is more concerned with time competition and will offer a shorter delivery time. Thus, the increased price is intended to enhance its profit. Result (ii) indicates that as the price attraction factor increases, the firm focuses on competing on price; thus, a lower price and longer delivery time can augment demand. Increasing the parameters that can reduce the cost of the delivery time leads to a longer delivery time, and the firm therefore reduces its price to lure more customers.

## Numerical experiments

In this section, a set of numerical experiments are conducted to understand the behaviour of the marketing controlled variables, the delivery time as well as the additional variable of price, and the operations controlled variable, order quantity, as depicted above. It is observed that some more general results draw from numerical experiments can be applicable in practice even if we do not present in the theoretical analysis. In order to better present the numerical experiments, we select the thriopoly of three interactive competitive firms 1, 2 and 3 as the setting of competition, because “three” is the minimum number in the oligopoly and still can confirm our results. It is intuitive that considering more rivals is not necessarily to offer the enough insights instead justify the additional complexity.

The ranges of each parameter and five levels are assigned in Table 3. The ranges of parameters *γ*, *c*_*h*_, and *c*_*o*_ for all rivals are determined by analysing the brewery and soft drink industries in the US [49]. The ranges of parameters *β* and *b* are influenced by the numerical results of So (2000) [22]. The other parameters, *δ* and *ϵ*, range from 100 to 500 and from 1 to 1.8, respectively, and the market potential *m* is equal to the constant 1,000. If we were to discuss all different parametric values, there exist 5^21^ cases for equilibrium, and it would be difficult for a typical computer to obtain all cases. Therefore, we will employ the fractional factorial design developed by Kutner et al. (2005) [50]. All model parameters are classified into two groups: marketing factors, including *γ*_*j*_, *β*_*j*_, *b*_*j*_, *δ*_*j*_ and *ϵ*_*j*_, and operations factors, including *C*_*hj*_ and *C*_*oj*_. Each group of parameters can be stratified into three levels: low, medium or high. Therefore, 3^2^ = 9 basic configurations of parameter assignments are shown in Table 4.

**Table 3:** Assigned parameter levels for a triopoly.

Parameters	Levels				
*γ*_1_, *γ*_2_, *γ*_3_	2	2.25	2.5	2.75	3
*β*_1_, *β*_2_, *β*_3_	0.6	0.825	1.05	1.275	1.5
*b*_1_, *b*_2_, *b*_3_	0.3	0.325	0.35	0.375	0.4
*δ*_1_, *δ*_2_, *δ*_3_	100	200	300	400	500
*ϵ*_1_, *ϵ*_2_, *ϵ*_3_	1	1.2	1.4	1.6	1.8
*C*_*h*1_, *C*_*h*2_, *C*_*h*3_	0.2	0.225	0.25	0.275	0.3
*C*_*o*1_, *C*_*o*2_, *C*_*o*3_	1	2.75	4.5	6.25	8

**Table 4:** Fractional factorial design for a non-dominated triopoly.

L-H			M-H			H-H		
Firm1	Firm2	Firm3	Firm1	Firm2	Firm3	Firm1	Firm2	Firm3
2	2.25	2.5	2.25	2.5	2.75	2.5	2.75	3
0.6	0.825	1.05	0.825	1.05	1.275	1.05	1.275	1.5
0.3	0.325	0.35	0.325	0.35	0.375	0.35	0.375	0.4
100	200	300	200	300	400	300	400	500
1	1.2	1.4	1.2	1.4	1.6	1.4	1.6	1.8
0.25	0.275	0.3	0.25	0.275	0.3	0.25	0.275	0.3
4.5	6.25	8	4.5	6.25	8	4.5	6.25	8
L-M			M-M			H-M		
Firm1	Firm2	Firm3	Firm1	Firm2	Firm3	Firm1	Firm2	Firm3
2	2.25	2.5	2.25	2.5	2.75	2.5	2.75	3
0.6	0.825	1.05	0.825	1.05	1.275	1.05	1.275	1.5
0.3	0.325	0.35	0.325	0.35	0.375	0.35	0.375	0.4
100	200	300	200	300	400	300	400	500
1	1.2	1.4	1.2	1.4	1.6	1.4	1.6	1.8
0.225	0.25	0.275	0.225	0.25	0.275	0.225	0.25	0.275
2.75	4.5	6.25	2.75	4.5	6.25	2.75	4.5	6.25
L-L			M-L			H-L		
Firm1	Firm2	Firm3	Firm1	Firm2	Firm3	Firm1	Firm2	Firm3
2	2.25	2.5	2.25	2.5	2.75	2.5	2.75	3
0.6	0.825	1.05	0.825	1.05	1.275	1.05	1.275	1.5
0.3	0.325	0.35	0.325	0.35	0.375	0.35	0.375	0.4
100	200	300	200	300	400	300	400	500
1	1.2	1.4	1.2	1.4	1.6	1.4	1.6	1.8
0.2	0.225	0.25	0.2	0.225	0.25	0.2	0.225	0.25
1	2.75	4.5	1	2.75	4.5	1	2.75	4.5

L: low; M: medium; H: high.

We first study Proposition 1 by employing the parameters depicted in Table 4. The equations for the equilibrium t*, q*, TC* and π* in an asymmetric decentralized oligopoly are obtained from Eqs (10), (11), (12) and (14) in the Appendix, respectively. Similarly, for an asymmetric centralized oligopoly, the equilibrium t**, q**, TC** and π** are obtained from Eqs (18), (20) and (21) in the Appendix, respectively. Therefore, the experimental results, which are in accord with the statements of Proposition 1, are reported in Table 5. The centralized delivery time is always longer than the decentralized delivery time, and the total profit is the same with the delivery times. However, $q_{j}^{**}$ ($TC_{j}^{**}$) is larger (smaller) than $q_{j}^{*}$ ($TC_{j}^{**}$) in some cases.

**Table 5:** Comparison of centralized and decentralized scenarios.

	Firm1	Firm2	Firm3		Firm1	Firm2	Firm3
Parameters					Measure		
*γ*	2	2.25	2.5	*t* *	0.8237	1.3933	1.6507
*β*	0.6	0.825	1.05	*t* **	0.8311	1.4095	1.6690
*b*	0.3	0.325	0.35	*Q* *	53.0734	89.5525	118.5277
*δ*	100	200	300	*Q* **	53.0948	89.5408	118.5063
*ϵ*	1	1.2	1.4	*TC* *	10.6147	20.1493	29.6319
*C*_*h*_	0.2	0.225	0.25	*TC* **	10.6190	20.1467	29.6266
*C*_*o*_	1	2.75	4.5	*π* *	431.3406	583.6913	797.2592
*m*	1000	1000	1000	*π* **	432.8746	585.3523	799.1896

*: decentralization scenario;

**: centralization scenario.

Secondly, we explore the advantage of cooperative competition. An illustrative example shows the role of cooperative competition. Let $f\left(t_{j}\right)=\beta_{j}{\left(\alpha_{j}+t_{j}\right)}^{-b_{j}}$ for all *j* = 1, 2…*n*, where *α*_*j*_ is such that the denominator of *f*(*t*_*j*_) is not zero. To gain a better understanding of the interaction effect between non-cooperative centralized competition and cooperative competition, two scenarios are considered: Scenario 1 is symmetric, whereas Scenario 2 is asymmetric. The results shown in Table 6 are consistent with Propositions 5 and 6. In Scenario 1, no firm will adopt the delivery time strategy because *CS*_1_ = *CS*_2_ = *CS*_3_ = *WMS*. In Scenario 2, only firm 3 will adopt such a strategy for *CS*_3_ > *WMS*. Additionally, the profits under cooperative competition are always larger than the those under asymmetric centralized competition, regardless of whether the competition is symmetric or asymmetric. The findings indicate that the mode of competition plays an essential role in determining whether a firm will adopt a suitable strategy for employing delivery time.

**Table 6:** Impact of cooperative competition.

	Scenario 1			Scenario 2		
	Firm 1	Firm 2	Firm 3	Firm 1	Firm 2	Firm 3
*α*_*j*_	0.1	0.1	0.1	0.2	0.3	0.4
*γ*_*j*_	2	2.0	2	2	2.5	3
*β*_*j*_	0.6	0.6	0.6	0.6	0.9	1.5
*b*_*j*_	0.3	0.3	0.3	0.3	0.35	0.4
*δ*_*j*_	500.0	500.0	500.0	100.0	200.0	300.0
*ϵ*_*j*_	1	1	1	1.0	1.2	1.4
*C*_*hj*_	0.2	0.2	0.2	0.2	0.225	0.25
*C*_*oj*_	1.0	1.0	1.0	1.0	2.75	4.5
*m*	1000.0	1000.0	1000.0	1000.0	1000.0	1000.0
Optimal solution						
$t_{j}^{**}$	3.7828	3.7828	3.7828	0.9964	1.2686	1.2827
$t_{j}^{***}$	S.L.	S.L.	S.L.	S.L.	S.L.	5.8780
$Q_{j}^{**}$	57.7350	57.7350	57.7350	46.4274	85.2304	132.4460
$Q_{j}^{***}$	57.7350	57.7350	57.7350	23.8559	38.4466	178.2535
$TC_{j}^{**}$	11.5470	11.5470	11.5470	9.2855	19.1768	33.1115
$TC_{j}^{***}$	11.5470	11.5470	11.5470	4.7712	8.6505	44.5634
$\pi_{j}^{**}$	522.9410	522.9410	522.9410	321.4552	573.4255	1216.9975
$\Sigma_{i=1}^{3}\pi_{jcor}^{**}$	1568.8230			2111.8782		
$\pi_{j}^{***}$	653.0096	653.0096	653.0096	109.0297	142.5139	2580.3793
$\pi_{cc}^{***}$	1959.02874			2831.9229		
*CS*_*j*_	1.9827	1.9827	1.9827	1.9581	2.4285	2.9748
*WMS*	1.9827			2.8839		

S.L.: stands for “Sufficiently Large”.

### Sensitivity analysis

In a market that is characterized by dominated competition, a higher degree of centralization facilitates the creation of monopolies, which will not be approved by the antitrust authorities. Economists and antitrust agencies that have studied monopolistic markets over the long term report that monopolies can increase the price of goods, damage the interests of consumers and affect social and economic benefits [51]. Thus, most markets are based on non-dominated competition, especially markets with time-based competition. As a consequence, the following numerical experiments primarily consider the case of a non-dominated oligopoly with three rivals, and shed light on the effects of changes in various parameters on the decision variables.

We firstly investigate the impacts that a firm’s own parameters and its rivals’ parameters have on the equilibrium delivery time and order quantity. Employing each of the parameters and allowing them to increase over their respective ranges depicted in Table 3, Table 7 provides the signs of the self-sensitivity analysis and cross-sensitivity analysis of the equilibrium quantities $MS_{j}^{**}$, $t_{j}^{**}$, $q_{j}^{**}$, $TC_{j}^{**}$ and $\pi_{j}^{**}$ (where *j* = 1, 2, 3) under a non-dominated coordinated asymmetric oligopoly, which is in conformity with Propositions 2 and 4. For example, Table 7 implies that when parameter *γ*_1_ increases, $t_{1}^{**}$ decreases, whereas both $t_{2}^{**}$ and $t_{3}^{**}$ increase. Note that the sign of $\partial t_{j}^{**}/\partial b_{j}$ (*j* = 1, 2, 3) is unknown from a theoretical perspective, but Table 7 shows that the parameter *b*_*j*_ has an adverse effect on the equilibrium delivery time. From Table 7, we conclude that the directional impact of changes in the parameters of a firm *j* on its own decision variables ($t_{j}^{**}$, $q_{j}^{**}$) is the opposite of that on its competitors ($t_{i}^{**}$, $q_{i}^{**}$) (*i* ≠ *j*;*j* = 1, 2, 3).

**Table 7:** The sign of the sensitivity analysis for a non-dominated asymmetric oligopoly.

	*γ*_*j*_	*γ*_*i*_	*β*_*j*_	*β*_*i*_	*b*_*j*_	*b*_*i*_	*δ*_*j*_	*δ*_*i*_	*ϵ*_*j*_	*ϵ*_*i*_	*C*_*hj*_	*C*_*hi*_	*C*_*oj*_	*C*_*oi*_
*t*_*j*_	-	+	-	+	-	+	+	-	+	-	+	-	+	-
*M*_*j*_	+	-	+	-	+	-	-	+	-	+	-	+	-	+
*Q*_*j*_	+	-	+	-	+	-	-	+	-	+	-	+	+	+
*TC*_*j*_	+	-	+	-	+	-	-	+	-	+	+	+	+	+
*π*_*j*_	+	-	+	-	+	-	-	+	-	+	-	+	-	+

+: Increase; -: decrease; *i* ≠ *j*.

Even if Table 7 describes the direction of the changes, it does not provide their magnitudes. In what follows, selecting a parameter makes it possible to vary the parameter across its assigned range while keeping the remaining 14 parameters unchanged. Following Pannell (1997) [52], the sensitivity indices (*SIs*) are expressed by $\frac{\left(EM_{max}^{**}-EM_{min}^{**}\right)}{EM_{max}^{**}}$, where *EM* delegates the equilibrium quantities t**, M** q**, TC** and π**. Then, the average *SIs* summarized in Table 8 are obtained from nine different basic configurations of parameter assignments. Analysing the obtained results generates the following main conclusions in a non-dominated competitive setting: (i) Changes in a parameter of a firm have a greater impact on its own equilibrium measures than on those of its competitors. (ii) Changes in marketing parameters for a given firm have a substantial impact on the delivery time, market share and profit of all firms, whereas changes in the operations parameters only affect the firm’s own order quantity and total inventory cost. Therefore, the marketing parameters play a more important role in equilibrium measures than do the operations parameters.

**Table 8:** Average sensitivity indices under non-dominated triopoly.

	*t*_1_	*t*_2_	*t*_3_	*MS*_1_	*MS*_2_	*MS*_3_	*q*_1_	*q*_2_	*q*_3_	*TC*_1_	*TC*_2_	*TC*_3_	*π*_1_	*π*_2_	*π*_3_
*γ*_1_	0.33	0.01	0.0	0.09	0.04	0.04	0.04	0.02	0.02	0.05	0.02	0.02	0.4	0.04	0.04
*γ*_2_	0.02	0.28	0.01	0.04	0.08	0.04	0.02	0.04	0.02	0.02	0.04	0.02	0.05	0.4	0.04
*γ*_3_	0.02	0.02	0.24	0.04	0.04	0.07	0.02	0.02	0.03	0.02	0.02	0.03	0.05	0.05	0.4
*β*_1_	0.26	0.14	0.09	0.51	0.3	0.28	0.3	0.16	0.15	0.3	0.16	0.15	0.55	0.32	0.31
*β*_2_	0.17	0.25	0.07	0.29	0.52	0.27	0.16	0.3	0.15	0.16	0.3	0.15	0.32	0.55	0.3
*β*_3_	0.15	0.1	0.21	0.29	0.28	0.51	0.16	0.15	0.3	0.16	0.15	0.3	0.32	0.3	0.55
*b*_1_	0.24	0.01	0.01	0.05	0.02	0.02	0.03	0.01	0.01	0.03	0.01	0.01	0.04	0.03	0.02
*b*_2_	0.01	0.19	0.0	0.01	0.02	0.01	0.01	0.01	0.0	0.01	0.01	0.0	0.01	0.04	0.01
*b*_3_	0.0	0.0	0.17	0.01	0.01	0.01	0.0	0.0	0.0	0.0	0.0	0.0	0.0	0.0	0.04
*δ*_1_	0.79	0.05	0.03	0.32	0.13	0.13	0.18	0.07	0.07	0.18	0.07	0.07	0.35	0.15	0.14
*δ*_2_	0.09	0.72	0.04	0.16	0.26	0.14	0.08	0.14	0.07	0.08	0.14	0.07	0.17	0.29	0.16
*δ*_3_	0.1	0.07	0.65	0.17	0.16	0.22	0.09	0.08	0.11	0.09	0.08	0.11	0.18	0.18	0.24
*ϵ*_1_	0.21	0.01	0.01	0.057	0.02	0.02	0.03	0.01	0.01	0.03	0.01	0.01	0.06	0.03	0.02
*ϵ*_2_	0.01	0.15	0.0	0.02	0.04	0.02	0.01	0.02	0.01	0.01	0.02	0.01	0.02	0.08	0.02
*ϵ*_3_	0.01	0.01	0.11	0.02	0.02	0.03	0.01	0.01	0.01	0.01	0.01	0.01	0.02	0.02	0.09
*c*_*h*1_	0.0	0.0	0.0	0.0	0.0	0.0	0.18	0.0	0.0	0.18	0.0	0.0	0.01	0.0	0.0
*c*_*h*2_	0.0	0.0	0.0	0.0	0.0	0.0	0.0	0.18	0.0	0.0	0.18	0.0	0.0	0.0	0.0
*c*_*h*3_	0.0	0.0	0.0	0.0	0.0	0.0	0.0	0.0	0.18	0.0	0.0	0.18	0.0	0.0	0.01
*c*_*o*1_	0.02	0.0	0.0	0.0	0.0	0.0	0.65	0.0	0.0	0.65	0.0	0.0	0.04	0.0	0.0
*c*_*o*2_	0.0	0.01	0.0	0.0	0.0	0.0	0.0	0.65	0.0	0.0	0.65	0.0	0.0	0.04	0.0
*c*_*o*3_	0.0	0.0	0.01	0.0	0.0	0.0	0.0	0.0	0.65	0.0	0.0	0.65	0.0	0.0	0.03

Table 9 summarizes how a given firm is affected by its optimal policy in response to changes in the model parameters when the additional price variable is incorporated into the profit-maximizing model under a non-dominated oligopoly. Although we only derive the symmetric results based on theory, we can extend the results to an asymmetric oligopoly by conducting numerical experiments, as illustrated in Table 9. We do not observe an aggregate effect of other factors on the optimal decision variables. Thus, we argue the situation where the inventory cost is negligible, as summarized in Table 9, which is congruous with the analytical findings from Proposition 9.

**Table 9:** Sensitivity analysis for a non-dominated competition with a price factor.

	Existence inventory			Loss of inventory	
	*t*_*j*_	*P*_*j*_	*Q*_*j*_	*t*_*j*_	*P*_*j*_
*MC*_*j*_	-	+	-	-	+
*β*_*j*_	N.O.	N.O.	N.O.	-	+
*a*_*j*_	+	-	+	+	-
*b*_*j*_	-	+	+	-	+
*δ*_*j*_	+	-	-	+	-
*ϵ*_*j*_	+	-	-	+	-
*C*_*hj*_	-	+	-		
*C*_*oj*_	-	+	+		

*N*.*O*.: stands for “Not Obtained”;

## Management implications

Our proposed delivery time and inventory model is applicable to the oligopoly market situations where the customers are sensitive to the delivery time and price of products. It can help all rivals to not only coordinate conflict of the marketing department and operations department for intra-firm competition, but also gain the directional impact of changes in marketing and operations factors on the optimal delivery times, prices and order quantities for inter-firm competition. In particular, many service firms and most online retailers operate under such a competition environment, and thus the results for the effects of marketing and operations factors on the optimal decisions can be easily adopted based on their oligopolistic structures.

In order to build the model for investigating the interactions between delivery time and inventory, we develop the market share attraction structure of delivery time competition by incorporating the traditional economic order quantity inventory management model, with delivery time and order quantity decisions being made by the marketing department and operations department, respectively. Firstly, we find that each decentralized firm has a critical inferiority over the centralized scenario due to the information asymmetry for intra-firm competition, even though the delivery time of each firm under decentralization is shorter. Hence, it is incentive for each decentralized firm to get coordinated in order to improve its profit in an asymmetric oligopolistic market. Secondly, by conducting analysis for inter-firm competition using a supermodular game, the results show that the optimal delivery time and order quantity of each firm exhibit a directional response with changes in marketing and operations factors, and the response in a non-dominated oligopoly differs from that in a dominated oligopoly. Consequently, each firm in oligopoly market has more flexibility in terms of changing its marketing and operations factors and justifies the directional effect of changes in marketing and operations factors on the optimal delivery and order quantity. In addition, it is found that all firms are favorable for the cooperative competition changed from noncooperation competition in an oligopolistic market. Moreover, for asymmetric oligopoly, firms with high competitive strength continue to adapting a delivery time strategy while the weak firms cease this strategy. On the other hand, none of firms selects to implement the delivery time strategy under a symmetric oligopoly market. Therefore, a category manager or a dominant company will rationalize the delivery time decisions based on their competitive strength.

Considering that delivery time and price are interdependent, the market share attraction structure is generalized to simultaneously deal with delivery time and price. Therefore, by adopting a similar supermodular game method, the results show that the firm in a non-dominated or dominated oligopolistic market should adapt different normative behaviors in response to the effects of the marketing and operations factors on the optimal delivery time, price and order quantity. Finally, the corresponding numerical experiments examine the effects of changes in marketing and operations factors on the marketing decision variables, namely delivery time and price, and the operations decision variable, namely order quantity. The numerical results reveal that the market factors studied have a greater impact on the examined equilibrium indicators than the considered operational factors. Firms in an oligopoly market should focus more on the value of marketing-oriented factors than operations factors.

## Conclusion

In contemporary competitive markets, timely deliver is considered an important factor. Many service companies employ a uniform delivery time for their products to compete for customers in a time-sensitive market. Using a stylized approach, the first main objective of this study is to analyse how a firm adjusts its optimal delivery time and the size of its order quantity in response to changes in its own relevant parameters in asymmetric non-dominated and dominated competitive settings using a supermodular game approach (Propositions 2 and 3). Next, the competition is confined to a symmetric duopoly model where we can derive the cross-sensitivity analysis (Proposition 4). The second main objective is to examine how changing the competitive mode from non-cooperation to cooperation affects the delivery time strategies (Propositions 5 and 6). The third main objective is to discuss how changes in all the model parameters affect the optimal responses of a given firm when price is considered as an additional marketing variable (Propositions 7, 8 and 9).

From a theoretical perspective, this paper employs a supermodular game approach as a novel application for the intersection of marketing and operations. The method, in essence, analyses the existence of a Nash equilibrium and provides a corresponding sensitivity analysis. Therefore, when employing an MCI market share model based on a delivery time, the optimal behaviours of all rivals in response to changes in the model parameters are asymmetric in a non-dominated oligopoly. However, the optimal behaviours of firms in a dominated oligopoly are instead symmetric. Next, we apply a similar method to discuss an extended market share model based on time and price. From a numerical perspective, Table 7 provides interesting insights for decision makers. We find that marketing parameters play a more important role in maximizing profits than do operations parameters. As a result, we obtained significant practical implications for a firm’s decision makers, namely, that they should focus on the values of marketing-oriented parameters rather than attempting to obtain information from operations parameters.

The studied model offers a framework for further research. The present study considers a classical EOQ model. Future research could be enriched by introducing shortage costs, lead times, taxes and inflation into the model. In this work, our results are illustrated using numerical simulations. Therefore, we can investigate the linkage between our findings and empirical data from several industries. Moreover, relaxing the deterministic market share attraction model of time competition and incorporating a stochastic mechanism would provide a plausible direction for future research.

## Appendix

### Proof of Proposition 1

In the decentralized case, the marketing department of firm *j* seeks to maximize revenue per unit time expressed by Eq (4), whereas the operations department of firm *j* seeks to minimize the total inventory cost per unit time expressed by Eq (3). At optimality, we obtain

$$\frac{\partial \pi_{jdec}^{r}}{\partial t_{j}}=\gamma_{j}\frac{mf_{j}^{{}^{\prime }}\sum_{i\neq j}\;f_{i}}{{\left(f_{j}+\sum_{i\neq j}\;f_{i}\right)}^{2}}+\delta_{j}\epsilon_{j}t_{j}^{-\epsilon_{j}-1}=0,\;\;\;j=1,2,...,n$$ (10)

Based on Gallego et al. (2006) [53], we can verify that the non-linear Eq (10) has a unique solution given by the row vector ${\vec{t}}^{*}=\left(t_{1}^{*},t_{2}^{*},...,t_{n}^{*}\right).$ It follows from the necessary condition for minimizing (3) that

$$Q_{j}^{*}=\sqrt{\frac{2C_{oj}}{C_{hj}}\frac{mf_{j}^{*}}{\sum_{i=1}^{n}f_{i}^{*}}}.$$ (11)

Substituting (11) into (3) yields

$$TC_{j}^{*}=\sqrt{2C_{oj}C_{hj}\frac{mf_{j}^{*}}{\sum_{i=1}^{n}f_{i}^{*}}}.$$ (12)

Since the sum of the market shares equals one, i.e., $\sum_{j=1}^{n}M_{j}=1$, we can summarize that the equilibrium order quantity $Q_{j}^{*}$ and the total inventory cost $TC_{j}^{*}$ can be depicted by

$$\frac{C_{hj}}{2mC_{oj}}\sum_{j=1}^{n}Q_{j}^{*2}=1,\frac{\sum_{j=1}^{n}TC_{j}^{*2}}{2mC_{oj}C_{hj}}=1.$$ (13)

Combining (2) and (12), the optimal decentralized profit is given by

$$\pi_{jdec}^{*}=\left(\gamma_{j}-\frac{C_{oj}}{Q_{j}^{*}}\right)\frac{mf_{j}\left(t_{j}^{*}\right)}{\sum_{i=1}^{n}f_{i}\left(t_{i}^{*}\right)}-C\left(t_{j}^{*}\right)-\frac{Q_{j}^{*}}{2}C_{hj}-F_{j},\;\;\;\;j=1,2,...,n.$$ (14)

In the centralized or coordinated case, the profit function of a coordinated firm *j* takes the following form:

$$\pi_{jcor}=\left(\gamma_{j}-\frac{C_{oj}}{Q_{j}}\right)\frac{mf_{j}\left(t_{j}\right)}{\sum_{i=1}^{n}f_{i}\left(t_{i}\right)}-\delta_{j}t_{j}^{-\epsilon_{j}}-\frac{Q_{j}}{2}C_{hj}-F_{j},\;\;\;\;j=1,2,...,n.$$ (15)

The two necessary conditions for maximizing (15) are given by

$$\begin{array}{c} \begin{array}{cc} & \frac{\partial \pi_{jcor}}{\partial t_{j}}=\left(\gamma_{j}-\frac{C_{oj}}{Q_{j}}\right)\frac{mf_{j}^{{}^{\prime }}\sum_{i\neq j}\;f_{i}}{{\left(f_{j}+\sum_{i\neq j}\;f_{i}\right)}^{2}}+\delta_{j}\epsilon_{j}t_{j}^{-\epsilon_{j}-1}=0,\\ & \frac{\partial \pi_{jcor}}{\partial Q_{j}}=\frac{mC_{oj}}{Q_{j}^{2}}\frac{f_{j}}{\sum_{i=1}^{n}f_{i}}-\frac{C_{hj}}{2}=0. \end{array} \end{array}$$ (16)

Note that the sufficiency of profit maximization must satisfy $\frac{\partial^{2}\pi_{jcor}}{\partial t_{j}^{2}}\leq 0$ and $\frac{\partial^{2}\pi_{jcor}}{\partial t_{j}^{2}}\frac{\partial^{2}\pi_{jcor}}{\partial Q_{j}^{2}}-{\left(\frac{\partial^{2}\pi_{jcor}}{\partial t_{j}\partial Q_{j}}\right)}^{2}\geq 0$. From the second equation of (16), this can be solved by

$$Q_{j}=\sqrt{\frac{2C_{oj}}{C_{hj}}\frac{mf_{j}}{\sum_{i=1}^{n}f_{i}}}.$$ (17)

Substituting (17) into the first equation of (16) produces

$$\frac{mf_{j}^{{}^{\prime }}\sum_{i\neq j}\;f_{i}}{{\left(f_{j}+\sum_{i\neq j}\;f_{i}\right)}^{2}}\left(\gamma_{j}-\frac{1}{2}\nu_{j}\right)+\delta_{j}\epsilon_{j}t_{j}^{-\epsilon_{j}-1}=0,$$ (18)

where $\nu_{j}=\sqrt{\frac{2C_{oj}C_{hj}}{mM_{j}}}$. Next, we can solve the nonlinear equations (18), producing the row vector ${\vec{t}}^{**}=\left(t_{1}^{**},t_{2}^{**},...,t_{n}^{**}\right)$, such that ${\vec{t}}^{**}>{\vec{t}}^{*}$ for all *j*. Considering (10) and evaluating $\frac{\pi_{jcor}}{\partial t_{j}}$ at $t_{j}^{*}$ results in

$$\frac{\pi_{jcor}}{\partial t_{j}}{|}_{t_{j}^{*}}=-\frac{\nu_{j}}{2}\frac{mf_{j}^{{}^{\prime }}\left(t_{j}^{*}\right)}{\sum_{i=1}^{n}f_{i}\left(t_{i}^{*}\right)},$$ (19)

which is positive for all *j* = 1, 2, …, *n*. Therefore, there is an incentive for each firm *j* to prolong its delivery time upon coordination; thus, part (i) of Proposition 1 is completely proved. Substitute the solution $t_{j}^{**}$ into (17). The importance of the total inventory cost is given by

$$\frac{C_{hj}}{2mC_{oj}}\sum_{j=1}^{n}Q_{j}^{**2}=1,\frac{\sum_{j=1}^{n}TC_{j}^{**2}}{2mC_{oj}C_{hj}}=1.$$ (20)

Examining (13) and (20) proves parts (ii) and (iii) of the proposition. The optimal coordinated profit is given by

$$\pi_{jcor}^{**}=\left(\gamma_{j}-\frac{C_{oj}}{Q_{j}^{**}}\right)\frac{mf_{j}\left(t_{j}^{**}\right)}{\sum_{i=1}^{n}f_{i}\left(t_{i}^{**}\right)}-C\left(t_{j}^{**}\right)-\frac{Q_{j}^{**}}{2}C_{hj}-F_{j},\;\;\;\;j=1,2,...,n.$$ (21)

Applying the mean value theorem to expression (21) in the neighbourhood of ${\vec{t}}^{*}$ yields

$$\pi_{jcor}^{**}=\pi_{jdec}^{*}+\sum_{i=1}^{n}\left(t_{j}^{**}-t_{j}^{*}\right)\frac{\partial \pi_{jcor}}{\partial t_{j}}{|}_{\vec{x}},$$ (22)

where the vector $\vec{x}={\vec{t}}^{*}+\lambda \left({\vec{t}}^{**}-{\vec{t}}^{*}\right),0<\lambda <1.$ Therefore, $\pi_{jcor}^{**}>\pi_{jdec}^{*}$ for all *j*, thus proving part (iv) of the proposition.

### Proof of Proposition 2

For a non-dominated asymmetric oligopoly, we will prove how the equilibrium behaves following changes in various model parameters by applying Lemma 1. Conditions (ii) and (iii) are satisfied by setting $t_{j}^{{}^{\prime }}=-t_{j}^{-b_{j}}$, $t_{i}^{{}^{\prime }}=t_{i}^{-b_{i}}$ and $Q_{j}^{{}^{\prime }}=-Q_{j}$ for all *j*. Then expression (15) is expressed as follows:

$$\pi_{j}=\left(\gamma_{j}+\frac{C_{oj}}{Q_{j}^{{}^{\prime }}}\right)\frac{-m\beta_{j}t_{j}^{{}^{\prime }}}{-\beta_{j}t_{j}^{{}^{\prime }}+\sum_{i\neq j}\beta_{i}t_{i}^{{}^{\prime }}}-\delta_{j}{\left(-t_{j}^{{}^{\prime }}\right)}^{\frac{\epsilon_{j}}{b_{j}}}+\frac{Q_{j}}{2}C_{hj}-F_{j}.$$

Let $A_{j}=-\beta_{j}t_{j}^{{}^{\prime }},A_{i}=\beta_{i}t_{i}^{{}^{\prime }}\left(i\neq j\right).$ Then,

$$\begin{array}{c} \begin{array}{cc} & \frac{\partial^{2}\pi_{j}}{\partial t_{j}^{{}^{\prime }}\partial Q_{j}^{{}^{\prime }}}=\frac{C_{oj}}{Q_{j}^{{}^{\prime }2}}\frac{m\beta_{j}\sum_{i\neq j}A_{i}}{{\left(A_{j}+\sum_{i\neq j}A_{i}\right)}^{2}}>0,\;\;\frac{\partial^{2}\pi_{j}}{\partial t_{j}^{{}^{\prime }}\partial t_{i}^{{}^{\prime }}}=\left(\gamma_{j}+\frac{C_{oj}}{Q_{j}^{{}^{\prime }}}\right)\frac{-m\beta_{j}\beta_{i}\left(A_{j}-\sum_{i\neq j}A_{i}\right)}{{\left(A_{j}+\sum_{i\neq j}A_{i}\right)}^{3}}>0,\\ & \frac{\partial^{2}\pi_{j}}{\partial t_{j}^{{}^{\prime }}\partial Q_{i}}=0,\;\;\frac{\partial^{2}\pi_{j}}{\partial Q_{j}^{{}^{\prime }}\partial t_{i}^{{}^{\prime }}}=\frac{C_{oj}}{Q_{j}^{{}^{\prime }2}}\frac{mA_{j}\beta_{i}}{{\left(A_{j}+\sum_{i\neq j}A_{i}\right)}^{2}}>0,\;\;\frac{\partial^{2}\pi_{j}}{\partial Q_{j}^{{}^{\prime }}\partial Q_{i}}=0. \end{array} \end{array}$$

For the parameter *θ* = *γ*_*j*_, by setting $\gamma_{j}^{{}^{\prime }}=-\gamma_{j}$, condition (iv) is satisfied as

$$\begin{array}{c} \frac{\partial^{2}\pi_{j}}{\partial t_{j}^{{}^{\prime }}\partial \gamma_{j}^{{}^{\prime }}}=\frac{m\beta_{j}\sum_{i\neq j}A_{i}}{{\left(A_{j}+\sum_{i\neq j}A_{i}\right)}^{2}}>0,\frac{\partial^{2}\pi_{j}}{\partial Q_{j}^{{}^{\prime }}\partial \gamma_{j}^{{}^{\prime }}}=0. \end{array}$$

Therefore, $\frac{\partial t_{j}^{{}^{\prime }}}{\partial \gamma_{j}^{{}^{\prime }}}\geq 0,\frac{\partial Q_{j}^{{}^{\prime }}}{\partial \gamma_{j}^{{}^{\prime }}}\geq 0$, so that $\frac{\partial t_{j}}{\partial \gamma_{j}}\leq 0,\frac{\partial Q_{j}}{\partial \gamma_{j}}\geq 0$.

For the parameter *θ* = *m*, by setting *m*′ = -*m*′, condition (iv) is satisfied as

$$\frac{\partial^{2}\pi_{j}}{\partial t_{j}^{{}^{\prime }}\partial \partial m^{{}^{\prime }}}=\left(\gamma_{j}+\frac{C_{oj}}{Q_{j}^{{}^{\prime }}}\right)\frac{\beta_{j}\sum_{i\neq j}A_{i}}{{\left(A_{j}+\sum_{i\neq j}A_{i}\right)}^{2}}>0,\frac{\partial^{2}\pi_{j}}{\partial Q_{j}^{{}^{\prime }}\partial m^{{}^{\prime }}}=\frac{C_{oj}}{Q_{j}^{{}^{\prime }2}}\frac{A_{j}}{A_{j}+\sum_{i\neq j}A_{i}}\geq 0.$$

Therefore, $\frac{\partial t_{j}^{{}^{\prime }}}{\partial m^{{}^{\prime }}}\geq 0,\frac{\partial Q_{j}^{{}^{\prime }}}{\partial m^{{}^{\prime }}}\geq 0$, so that $\frac{\partial t_{j}}{\partial m}\leq 0,\frac{\partial Q_{j}}{\partial m}\geq 0$.

For the parameter *θ* = *β*_*j*_, by setting $\beta_{j}^{{}^{\prime }}=-\beta_{j}$, condition (iv) is satisfied as

$$\begin{array}{c} \begin{array}{cc} & \frac{\partial^{2}\pi_{j}}{\partial t_{j}^{{}^{\prime }}\partial \beta_{j}^{{}^{\prime }}}=\left(\gamma_{j}+\frac{C_{oj}}{Q_{j}^{{}^{\prime }}}\right)\frac{m\left(\sum_{i\neq j}A_{i}\right)\left(\sum_{i\neq j}A_{i}-A_{j}\right)}{{\left(A_{j}+\sum_{i\neq j}A_{i}\right)}^{3}}>0,\\ & \frac{\partial^{2}\pi_{j}}{\partial Q_{j}^{{}^{\prime }}\partial \beta_{j}^{{}^{\prime }}}=-\frac{C_{oj}}{Q_{j}^{{}^{\prime }2}}\frac{mt_{j}^{{}^{\prime }}\sum_{i\neq j}A_{i}}{{\left(A_{j}+\sum_{i\neq j}A_{i}\right)}^{2}}>0 \end{array} \end{array}$$

Therefore, $\frac{\partial t_{j}^{{}^{\prime }}}{\partial \beta_{j}^{{}^{\prime }}}\geq 0,\frac{\partial Q_{j}^{{}^{\prime }}}{\partial \beta_{j}^{{}^{\prime }}}\geq 0$, so that $\frac{\partial t_{j}}{\partial \beta_{j}}\leq 0,\frac{\partial Q_{j}}{\partial \beta_{j}}\geq 0$.

For the parameter *θ* = *b*_*j*_, by setting $b_{j}^{{}^{\prime }}=-b_{j}$ condition (iv) is satisfied as

$$\frac{\partial^{2}\pi_{j}}{\partial t_{j}^{{}^{\prime }}\partial b_{j}^{{}^{\prime }}}=\frac{\delta_{j}\epsilon_{j}}{b_{j}^{{}^{\prime }2}}{\left(-t_{j}^{{}^{\prime }}\right)}^{\frac{\epsilon_{j}}{-b_{j}^{{}^{\prime }}}-1}\left[1+ln{\left(-t_{j}^{{}^{\prime }}\right)}^{\frac{\epsilon_{j}}{b_{j}}}\right]>0,\frac{\partial^{2}\pi_{j}}{\partial Q_{j}^{{}^{\prime }}\partial b_{j}^{{}^{\prime }}}=0.$$

Therefore, $\frac{\partial t_{j}^{{}^{\prime }}}{\partial b_{j}^{{}^{\prime }}}\geq 0,\frac{\partial Q_{j}^{{}^{\prime }}}{\partial b_{j}^{{}^{\prime }}}\geq 0$, so that $\frac{\partial t_{j}^{-b_{j}}}{\partial b_{j}}\geq 0,\frac{\partial Q_{j}}{\partial b_{j}}\geq 0$.

For the parameter *θ* = *δ*_*j*_, condition (iv) is satisfied as

$$\frac{\partial^{2}\pi_{j}}{\partial t_{j}^{{}^{\prime }}\partial \delta_{j}}=-\frac{\epsilon_{j}}{b_{j}}{\left(-t_{j}^{{}^{\prime }}\right)}^{\frac{\epsilon_{j}}{b_{j}}-1}\left(-1\right)>0,\frac{\partial^{2}\pi_{j}}{\partial Q_{j}^{{}^{\prime }}\partial \delta_{j}}=0.$$

Therefore, $\frac{\partial t_{j}^{{}^{\prime }}}{\partial \delta_{j}}\geq 0,\frac{\partial Q_{j}^{{}^{\prime }}}{\partial \delta_{j}}\geq 0$, so that $\frac{\partial t_{j}}{\partial \delta_{j}}\geq 0,\frac{\partial Q_{j}}{\partial \delta_{j}}\leq 0$.

For the parameter *θ* = *ϵ*_*j*_, condition (iv) is satisfied as

$$\frac{\partial^{2}\pi_{j}}{\partial t_{j}^{{}^{\prime }}\partial \epsilon_{j}}=\frac{\delta_{j}}{b_{j}}{\left(-t_{j}^{{}^{\prime }}\right)}^{\frac{\epsilon_{j}}{b_{j}}-1}\left[1+ln{\left(-t_{j}^{{}^{\prime }}\right)}^{\frac{\epsilon_{j}}{b_{j}}}\right]>0,\frac{\partial^{2}\pi_{j}}{\partial Q_{j}^{{}^{\prime }}\partial \epsilon_{j}}=0.$$

Therefore, $\frac{\partial t_{j}^{{}^{\prime }}}{\partial \epsilon_{j}}\geq 0,\frac{\partial Q_{j}^{{}^{\prime }}}{\partial \epsilon_{j}}\geq 0$, so that $\frac{\partial t_{j}}{\partial \epsilon_{j}}\geq 0,\frac{\partial Q_{j}}{\partial \epsilon_{j}}\leq 0$.

For the parameter *θ* = *C*_*hj*_, condition (iv) is satisfied as

$$\frac{\partial^{2}\pi_{j}}{\partial t_{j}^{{}^{\prime }}\partial C_{hj}}=0,\frac{\partial^{2}\pi_{j}}{\partial Q_{j}^{{}^{\prime }}\partial C_{hj}}=\frac{1}{2}>0.$$

Therefore, $\frac{\partial t_{j}^{{}^{\prime }}}{\partial C_{hj}}\geq 0,\frac{\partial Q_{j}^{{}^{\prime }}}{\partial C_{hj}}\geq 0$, so that $\frac{\partial t_{j}}{\partial C_{hj}}\geq 0,\frac{\partial Q_{j}}{\partial C_{hj}}\leq 0$.

### Proof of Proposition 3

The proof resembles that of Proposition 2 for a dominated asymmetric oligopoly. First, conditions (ii) and (iii) of Lemma 1 are satisfied by setting $t_{j}^{{}^{\prime }}=t_{j}^{-b_{j}}$,$t_{i}^{{}^{\prime }}=-t_{i}$, and $Q_{j}^{{}^{\prime }}=-Q_{j}$ for all *j*. By direct calculation, condition (iv) is satisfied as *θ* = *β*_*j*_, *δ*_*j*_, *ϵ*_*j*_ or *C*_*oj*_. Second, for parameters *γ*_*j*_ and *C*_*hj*_, by setting $\gamma_{j}^{{}^{\prime }}=-\gamma_{j}$ and $C_{hj}^{{}^{\prime }}=-C_{hj}$, the condition (iv) can be realized. Finally, for parameter *b*_*j*_, we should reset the change in the variables, letting $t_{i}^{{}^{\prime }}=-t_{i}$. Then, all of the conditions of Lemma 1 are satisfied.

### Proof of Proposition 4

The elements of matrix *H* are obtained by partially differentiating Eq (2) under a symmetric duopoly. First, the symmetric optimal solutions are obtained from the following expressions:

$$\frac{\partial \pi }{\partial t}=\left(\gamma -\frac{C_{o}}{Q}\right)\frac{-mb}{4t}+\delta \epsilon t^{-\epsilon -1}=0,\frac{\partial \pi }{\partial Q}=\frac{mC_{o}}{2Q^{2}}-\frac{C_{h}}{2}=0.$$

Solving the equations yields

$$t^{**}={\left(\frac{\gamma -mb\sqrt{C_{h}C_{o}}}{4\delta \epsilon }\right)}^{-\frac{1}{\epsilon }},Q^{**}=\sqrt{\frac{mC_{o}}{C_{h}}}.$$

Therefore, the elements of matrix *H* can be simplified based on the following definitions:

$$\begin{array}{c} \begin{array}{cc} & X=\frac{\partial^{2}\pi_{1}}{\partial t_{1}\partial Q_{1}}=\frac{\partial^{2}\pi_{2}}{\partial t_{2}\partial Q_{2}}=-\frac{mC_{o}}{Q^{2}}\frac{b}{4t}<0,\\ & Y=\frac{\partial^{2}\pi_{1}}{\partial t_{1}^{2}}=\frac{\partial^{2}\pi_{2}}{\partial t_{2}^{2}}=\left(\gamma -\frac{C_{o}}{Q}\right)\frac{mb}{4t^{2}}-\delta \epsilon \left(\epsilon +1\right)t^{-\epsilon -2}=\left(\gamma -\frac{C_{o}}{Q}\right)\frac{-mb\epsilon }{4t^{2}}<0. \end{array} \end{array}$$ (23)

From (23), it can be easily shown that $\frac{\partial^{2}\pi_{j}}{\partial t_{j}^{2}}\frac{\partial^{2}\pi_{j}}{\partial Q_{j}^{2}}-{\left(\frac{\partial^{2}\pi_{j}}{\partial t_{j}\partial Q_{j}}\right)}^{2}=-\frac{YmC_{o}}{Q^{3}}-X^{2}>0$ for *j* = 1, 2. Matrix *H* (see expression (8) in the main text), therefore, takes the following form:

$$\begin{array}{c} H=(\begin{array}{cccc} Y & X & 0 & 0\\ X & -\frac{mC_{o}}{Q^{3}} & -X & 0\\ 0 & 0 & Y & X\\ -X & 0 & X & -\frac{mC_{o}}{Q^{3}} \end{array}). \end{array}$$

In the following, we elucidate that *H* is negative semi-definite by means of the *kth* principal minor determinant

$$\begin{array}{c} \begin{array}{cc} & \Delta_{11}=Y<0,\;\;\Delta_{22}=-\frac{YmC_{o}}{Q^{3}}-X^{2}=\Delta_{1}>0,\;\;\Delta_{33}=Y\Delta_{22}<0,\\ & \Delta_{44}=\left|H\right|=\Delta_{1}^{2}-X^{4}=\left(\Delta_{1}-X^{2}\right)\left(\Delta_{1}+X^{2}\right)\geq 0\;if\;and\;only\;if\;\Delta_{1}-X^{2}\geq 0. \end{array} \end{array}$$

In light of $H^{-1}=\frac{H^{*}}{\left|H\right|}$, where H* is an adjugate matrix, we obtain

$$\begin{array}{c} H^{-1}=(\begin{array}{cccc} -\frac{mC_{o}}{Q^{3}\Delta_{2}} & -\frac{X}{\Delta_{2}} & \frac{X^{2}mC_{o}}{Q^{3}\Delta_{1}\Delta_{2}} & \frac{X^{3}}{\Delta_{1}\Delta_{2}}\\ -\left(1+\frac{X^{2}}{\Delta_{1}}\right)\frac{X}{\Delta_{2}} & \frac{Y}{\Delta_{2}} & -\frac{XYmC_{o}}{Q^{3}\Delta_{1}\Delta_{2}} & -\frac{X^{2}Y}{\Delta_{1}\Delta_{2}}\\ \frac{X^{2}mC_{o}}{Q^{3}\Delta_{1}\Delta_{2}} & \frac{X^{3}}{\Delta_{1}\Delta_{2}} & -\frac{mC_{o}}{Q^{3}\Delta_{2}} & -\frac{X}{\Delta_{2}}\\ -\frac{XYmC_{o}}{Q^{3}\Delta_{1}\Delta_{2}} & -\frac{X^{2}Y}{\Delta_{1}\Delta_{2}} & -\left(1+\frac{X^{2}}{\Delta_{1}}\right)\frac{X}{\Delta_{2}} & \frac{Y}{\Delta_{2}} \end{array}), \end{array}$$

where $\Delta_{1}=-\frac{YmC_{o}}{Q^{3}}-X^{2}>0$, $\Delta_{2}=\frac{\Delta_{1}^{2}-X^{4}}{\Delta_{1}}=\frac{\left(\Delta_{1}+X^{2}\right)\left(\Delta_{1}-X^{2}\right)}{\Delta_{1}}>0.$.

For the comparative statics of *γ*_1_, replacing *θ* with *γ*_1_, Eq (8) in the main text takes the following form:

$$\begin{array}{c} (\begin{array}{c} \frac{\partial t_{1}}{\partial \gamma_{1}}\\ \frac{\partial Q_{1}}{\partial \gamma_{1}}\\ \frac{\partial t_{2}}{\partial \gamma_{1}}\\ \frac{\partial Q_{2}}{\partial \gamma_{1}} \end{array})=-(\begin{array}{cccc} -\frac{mC_{o}}{Q^{3}\Delta_{2}} & -\frac{X}{\Delta_{2}} & \frac{mC_{o}X^{2}}{Q^{3}\Delta_{1}\Delta_{2}} & \frac{X^{3}}{\Delta_{1}\Delta_{2}}\\ -\left(1+\frac{X^{2}}{\Delta_{1}}\right)\frac{X}{\Delta_{2}} & \frac{Y}{\Delta_{2}} & -\frac{XYmC_{o}}{Q^{3}\Delta_{1}\Delta_{2}} & -\frac{X^{2}Y}{\Delta_{1}\Delta_{2}}\\ \frac{mC_{o}X^{2}}{Q^{3}\Delta_{1}\Delta_{2}} & \frac{X^{3}}{\Delta_{1}\Delta_{2}} & -\frac{mC_{o}}{Q^{3}\Delta_{2}} & -\frac{X}{\Delta_{2}}\\ -\frac{XYmC_{o}}{Q^{3}\Delta_{1}\Delta_{2}} & -\frac{X^{2}Y}{\Delta_{1}\Delta_{2}} & -\left(1+\frac{X^{2}}{\Delta_{1}}\right)\frac{X}{\Delta_{2}} & \frac{Y}{\Delta_{2}} \end{array})(\begin{array}{c} -\frac{mb}{4t}\\ 0\\ 0\\ 0 \end{array}). \end{array}$$

By computing matrix multiplications, the following results are given by

$$\begin{array}{c} \begin{array}{c} \frac{\partial t_{1}}{\partial \gamma_{1}}=-\frac{m^{2}bC_{o}}{4Q^{3}t\Delta_{2}}<0,\frac{\partial Q_{1}}{\partial \gamma_{1}}=-\left(1+\frac{X^{2}}{\Delta_{1}}\right)\frac{mbX}{4t\Delta_{2}}>0,\\ \frac{\partial t_{2}}{\partial \gamma_{1}}=\frac{X^{2}m^{2}bC_{o}}{4Q^{3}t\Delta_{1}\Delta_{2}}>0,\frac{\partial Q_{2}}{\partial \gamma_{1}}=-\frac{XYm^{2}bC_{o}}{4Q^{3}t\Delta_{1}\Delta_{2}}<0. \end{array} \end{array}$$

Similarly, the comparative statics of *β*_1_ can be given by

$$\begin{array}{c} \begin{array}{cc} & \frac{\partial t_{1}}{\partial \beta_{1}}=\frac{XmC_{o}}{4Q^{2}\beta \Delta_{2}}+\frac{X^{3}mC_{o}}{4Q^{2}\beta \Delta_{1}\Delta_{2}}<0,\frac{\partial Q_{1}}{\partial \beta_{1}}=-\frac{YmC_{o}}{4Q^{2}\beta \Delta_{2}}-\frac{X^{2}YmC_{o}}{4Q^{2}\beta \Delta_{1}\Delta_{2}}>0,\\ & \frac{\partial t_{2}}{\partial \beta_{1}}=-\frac{X^{3}mC_{o}}{4Q^{2}\beta \Delta_{1}\Delta_{2}}-\frac{XmC_{o}}{4Q^{2}\beta \Delta_{2}}>0,\frac{\partial Q_{2}}{\partial \beta_{1}}=\frac{X^{2}YmC_{o}}{4Q^{2}\beta \Delta_{1}\Delta_{2}}+\frac{YmC_{o}}{4Q^{2}\beta \Delta_{2}}<0. \end{array} \end{array}$$

The comparative statics of *b*_1_ can be given by

$$\begin{array}{c} \begin{array}{cc} & \frac{\partial t_{1}}{\partial b_{1}}=-\frac{(\Delta_{1}+X^{2})t}{\Delta_{2}b}\left(\frac{1}{\epsilon }+\frac{X^{2}lnt}{\Delta_{1}}\right)<0,\frac{\partial Q_{1}}{\partial b_{1}}=\frac{(\Delta_{1}+X^{2})XYt}{\Delta_{1}\Delta_{2}b\epsilon }\left(1-\epsilon lnt\right)>0,\\ & \frac{\partial t_{2}}{\partial b_{1}}=\frac{\left(\Delta_{1}+X^{2}\right)X^{2}t}{\Delta_{1}\Delta_{2}b\epsilon }\left(1-\epsilon lnt\right)>0,\frac{\partial Q_{2}}{\partial b_{1}}=-\frac{(\Delta_{1}+X^{2})XYt}{\Delta_{1}\Delta_{2}b\epsilon }\left(1-\epsilon lnt\right)<0. \end{array} \end{array}$$

The comparative statics of *δ*_1_ can be given by

$$\begin{array}{c} \begin{array}{cc} & \frac{\partial t_{1}}{\partial \delta_{1}}=\frac{mC_{o}\epsilon t^{-\epsilon -1}}{Q^{3}\Delta_{2}}>0,\frac{\partial Q_{1}}{\partial \delta_{1}}=\left(1+\frac{X^{2}}{\Delta_{1}}\right)\frac{X}{\Delta_{2}\epsilon t^{-\epsilon -1}}<0,\\ & \frac{\partial t_{2}}{\partial \delta_{1}}=-\frac{X^{2}mC_{o}\epsilon t^{-\epsilon -1}}{Q^{3}\Delta_{1}\Delta_{2}}<0,\frac{\partial Q_{2}}{\partial \delta_{1}}=\frac{XYmC_{o}\epsilon t^{-\epsilon -1}}{Q^{3}\Delta_{1}\Delta_{2}}>0. \end{array} \end{array}$$

The comparative statics of *ϵ*_1_ can be given by

$$\begin{array}{c} \begin{array}{cc} & \frac{\partial t_{1}}{\partial \epsilon_{1}}=\frac{mC_{o}\delta t^{-\epsilon -1}\left(1+lnt^{-\epsilon }\right)}{Q^{3}\Delta_{2}}>0,\frac{\partial Q_{1}}{\partial \epsilon_{1}}=\left(1+\frac{X^{2}}{\Delta_{1}}\right)\frac{X\delta t^{-\epsilon -1}\left(1+lnt^{-\epsilon }\right)}{\Delta_{2}}<0,\\ & \frac{\partial t_{2}}{\partial \epsilon_{1}}=-\frac{X^{2}mC_{o}\delta t^{-\epsilon -1}\left(1+lnt^{-\epsilon }\right)}{Q^{3}\Delta_{1}\Delta_{2}}<0,\frac{\partial Q_{2}}{\partial \epsilon_{1}}=\frac{XYmC_{o}\delta t^{-\epsilon -1}\left(1+lnt^{-\epsilon }\right)}{Q^{3}\Delta_{1}\Delta_{2}}>0. \end{array} \end{array}$$

The comparative statics of *C*_*h*1_ can be given by

$$\frac{\partial t_{1}}{\partial C_{h1}}=-\frac{X}{2\Delta_{2}}>0,\frac{\partial Q_{1}}{\partial C_{h1}}=\frac{Y}{2\Delta_{2}}<0,\frac{\partial t_{2}}{\partial C_{h1}}=\frac{X^{3}}{2\Delta_{1}\Delta_{2}}<0,\frac{\partial Q_{2}}{\partial C_{h1}}=\frac{X^{2}Y}{2\Delta_{1}\Delta_{2}}>0.$$

The comparative statics of *C*_*o*1_ can be given by

$$\begin{array}{c} \begin{array}{cc} & \frac{\partial t_{1}}{\partial C_{o1}}=-\frac{Xm}{2Q^{2}\Delta_{2}}>0,\;\left(using\;\left(23\right)\right),\\ & \frac{\partial Q_{1}}{\partial C_{o1}}=-\frac{Ym(\Delta_{1}-2X^{2})}{2Q^{2}\Delta_{1}\Delta_{2}}>0\left(<0\right)\;if\;\Delta_{1}-2X^{2}>\left(<\right)0,\\ & \frac{\partial t_{2}}{\partial C_{o1}}=\frac{X^{3}m}{2Q^{2}\Delta_{1}\Delta_{2}}<0,\;\left(using\;\left(23\right)\right),\\ & \frac{\partial Q_{2}}{\partial C_{o1}}=-\frac{X^{2}Ym}{2Q^{2}\Delta_{1}\Delta_{2}}>0,\;\left(using\;\left(23\right)\right). \end{array} \end{array}$$

### Proof of Proposition 5

Under cooperative competition, the total profit for the monopolist *π*_*cc*_ is given by

$$\pi_{cc}=\Sigma_{j=1}^{n}\pi_{jcor},$$ (24)

where *π*_*jcor*_ is obtained from an expression similar to (2). The two necessary conditions for maximizing (24) take the following form after minor rearrangements of the terms:

$$\begin{array}{c} \begin{array}{cc} & \frac{\partial \pi_{cc}}{\partial t_{j}}=\frac{mf_{j}^{{}^{\prime }}}{{\left(\sum_{i=1}^{n}f_{i}\right)}^{2}}\left[\left(\gamma_{j}-\frac{C_{oj}}{Q_{j}}\right)\sum_{i\neq j}f_{i}-\sum_{k\neq j}\left(\gamma_{k}-\frac{C_{ok}}{Q_{k}}\right)f_{k}\right]+\delta_{j}\epsilon_{j}t_{j}^{-\epsilon_{j}-1},\\ & \frac{\partial \pi_{cc}}{\partial Q_{j}}=\frac{mC_{oj}}{Q_{j}^{2}}\frac{f_{j}}{\sum_{i=1}^{n}f_{i}}-\frac{C_{hj}}{2}=0. \end{array} \end{array}$$ (25)

According to the second equation in (25), adding and subtracting the term $f_{j}\left(\gamma_{j}-\frac{\nu_{j}}{2}\right)$ to the bracket *[*.*]* in the first equation in (25) yields

$$\begin{array}{ccc} \frac{\partial \pi_{cc}}{\partial t_{j}} & = & \frac{mf_{j}^{{}^{\prime }}}{{\left(\sum_{i=1}^{n}f_{i}\right)}^{2}}\left[\left(\gamma_{j}-\frac{\nu_{j}}{2}\right)\sum_{i\neq j}f_{i}-\sum_{k\neq j}\left(\gamma_{k}-\frac{\nu_{k}}{2}\right)f_{k}\right]-\delta_{j}\left(-\epsilon_{j}\right)t_{j}^{-\epsilon_{j}-1}\\ & = & \frac{mf_{j}^{{}^{\prime }}}{\sum_{i=1}^{n}f_{i}}\left[\left(\gamma_{j}-\frac{\nu_{j}}{2}\right)-\sum_{k=1}M_{k}\left(\gamma_{k}-\frac{\nu_{k}}{2}\right)\right]+\delta_{j}\epsilon_{j}t_{j}^{-\epsilon_{j}-1}, \end{array}$$ (26)

where $\nu_{j}=\sqrt{\frac{2C_{oj}C_{hj}}{mM_{j}}}$. Next, we show that the solution of the nonlinear system (25) exists by denoting the row vector ${\vec{t}}_{j}^{***}=\left(t_{1}^{***},t_{2}^{***},...,t_{n}^{***}\right)$ for the monopolist such that ${\vec{t}}_{j}^{***}\geq {\vec{t}}_{j}^{**}$ under coordinated competition for all *j* = 1, 2, …, *n*. An evaluation of the first equation of (25) at the optimum vector ${\vec{t}}_{j}^{**}=\left(t_{1}^{**},t_{2}^{**},...,t_{n}^{**}\right)$ while employing (18) is given by

$$\frac{\partial \pi_{cc}}{\partial t_{j}}{|}_{{\vec{t}}_{j}^{**}}=-\frac{mf_{j}^{{}^{\prime }}}{\left(\sum_{i=1}^{n}f_{i}\right)}\left[\sum_{k=1}M_{k}\left(\gamma_{k}-\frac{\nu_{k}}{2}\right)\right]\geq 0,$$

which is a positive quantity for all *j* = 1, 2, …, *n*. Therefore, part (i) of Proposition 5 is completely proved. Substituting the solution $t_{j}^{***}$ into the second equation of (25), the equilibrium order quantity $Q_{j}^{***}$ lies on the surface of an n-dimensional ellipsoid

$$\frac{C_{hj}}{2mC_{oj}}\sum_{j=1}^{n}Q_{j}^{*2}=1,.$$

This proves part (ii) of Proposition 5. Substituting $t_{j}^{***}$ and $Q_{j}^{***}$ into (24), the optimal cooperative competition profit of the monopolist would be given by

$$\pi_{cc}^{***}=m\sum_{j=1}^{n}M_{j}^{***}\left(\gamma_{j}-\frac{\nu_{j}^{***}}{2}\right)-\sum_{j=1}^{n}C_{j}^{***}-\sum_{j=1}^{n}F_{j}^{***}.$$ (27)

Applying the mean value theorem to (27) in the neighbourhood of $t_{j}^{**}$ yields

$$\pi_{o\;mon}=\sum \pi_{j\;cor}^{**}+\sum \left(t_{j}^{***}-t_{j}^{**}\right)\frac{\partial \pi_{o\;mon}}{\partial t_{j}}{|}_{\vec{h}}>0,$$

where $\vec{h}={\vec{t_{j}}}^{**}+\phi \left({\vec{t_{j}}}^{***}-{\vec{t_{j}}}^{**}\right),0<\phi <1.$ Therefore, $\pi_{o\;mon}^{***}>\Sigma \pi_{j\;cor}^{**}$ and thus proving part (iii) of Proposition 5.

### Proof of Proposition 6

For the proof of Proposition 6, we only concentrate on the expression (26). First, note that if the market is characterized by weak cooperative competition for all firms, the sign of the terms inside the bracket *[*.*]* of expression of (26) is negative. As a result, expression (26) becomes positive, which implies that the monopoly market does not adopt the time guarantee strategy when maximizing profits. However, the market exhibits fierce cooperative competition for all firms, implying that the monopolist firm’s optimal response is to adopt a delivery time strategy on $t_{j}^{***}>0$ to obtain a higher profit. This completes the proof of Proposition 6.

### Proof of Proposition 7

For the profit of firm *j* shown below, we can derive the first- and second-order partial derivatives.

$$\pi_{j}=\left(P_{j}-MC_{j}-\frac{C_{oj}}{Q_{j}}\right)\frac{m\beta_{j}P_{j}^{-a_{j}}t_{j}^{-b_{j}}}{\sum_{i=1}^{n}\beta_{i}P_{j}^{-a_{i}}t_{i}^{-b_{i}}}-\delta_{j}t_{j}^{-\epsilon_{j}}-\frac{Q_{j}}{2}C_{hj}-F_{j},\;\;\;\;j=1,2,...,n.$$ (28)

Let $E_{j}=\beta_{j}P_{j}^{-a_{j}}t_{j}^{-b_{j}},E_{i}=\beta_{i}P_{j}^{-a_{i}}t_{i}^{-b_{i}}\left(i\neq j\right)$. Thus,

$$\begin{array}{c} \begin{array}{cc} & \frac{\partial \pi_{j}}{\partial t_{j}}=\left(P_{j}-MC_{j}-\frac{C_{oj}}{Q_{j}}\right)\frac{-m\beta_{j}b_{j}P_{j}^{-a_{j}}t_{j}^{-b_{j}-1}\sum_{i\neq j}E_{i}}{{\left(E_{j}+\sum_{i\neq j}^{n}E_{i}\right)}^{2}}+\delta_{j}\epsilon_{j}t_{j}^{-\epsilon_{j}-1},\\ & \frac{\partial \pi_{j}}{\partial P_{j}}=\frac{mE_{j}}{E_{j}+\sum_{i\neq j}^{n}E_{i}}\left[1+\left(P_{j}-MC_{j}-\frac{C_{oj}}{Q_{j}}\right)\frac{-a_{j}P_{j}^{-1}\sum_{i\neq j}E_{i}}{E_{j}+\sum_{i\neq j}^{n}E_{i}}\right],\\ & \frac{\partial \pi_{j}}{\partial Q_{j}}=\frac{mC_{oj}}{Q_{j}^{2}}\frac{E_{j}}{E_{j}+\sum_{i\neq j}^{n}E_{i}}-\frac{C_{hj}}{2}. \end{array} \end{array}$$ (29)

For symmetric competition, the equilibrium quantities t*, P*, Q* are obtained from (29), which is given by

$$\begin{array}{c} \begin{array}{cc} & t^{*}={\left(\frac{P^{*}mb}{na\delta \epsilon }\right)}^{-\frac{1}{\epsilon }},P^{*}=\left(MC+\sqrt{\frac{nC_{h}C_{o}}{2m}}\right)\frac{a(n-1)}{an-a-n}>0\;\left(\;if\;and\;only\;if\;a>\frac{n}{n-1}\right),\\ & Q^{*}=\sqrt{\frac{2mC_{o}}{nC_{h}}}. \end{array} \end{array}$$

Moreover, the second partial derivatives take the following forms:

$$\begin{array}{c} \begin{array}{cc} & \frac{\partial^{2}\pi_{j}}{\partial t_{j}^{2}}=-\frac{P^{*}mb}{nat^{*2}}\left(\frac{2b}{n}-b+\epsilon \right),\frac{\partial^{2}\pi_{j}}{\partial t_{j}\partial P_{j}}=-\frac{mb}{n^{2}t^{*}},\frac{\partial^{2}\pi_{j}}{\partial t_{j}\partial Q_{j}}=-\frac{C_{h}b\left(n-1\right)}{2nt^{*}},\\ & \frac{\partial^{2}\pi_{j}}{\partial P_{j}^{2}}=-\frac{m(a-1)}{nP^{*}},\frac{\partial^{2}\pi_{j}}{\partial P_{j}\partial Q_{j}}=-\frac{aC_{h}\left(n-1\right)}{2nP^{*}},\frac{\partial^{2}\pi_{j}}{\partial Q_{j}^{2}}=-\frac{C_{h}}{Q^{*}}. \end{array} \end{array}$$

Thus the Hessian matrix *H*_3×3_ of the second partial derivatives is shown below

$$\begin{array}{c} H=(\begin{array}{ccc} \frac{\partial^{2}\pi }{\partial t^{2}} & \frac{\partial^{2}\pi }{\partial t\partial P} & \frac{\partial^{2}\pi }{\partial t\partial Q}\\ \frac{\partial^{2}\pi }{\partial P\partial t} & \frac{\partial^{2}\pi }{\partial P^{2}} & \frac{\partial^{2}\pi }{\partial P\partial Q}\\ \frac{\partial^{2}\pi }{\partial Q\partial t} & \frac{\partial^{2}\pi }{\partial Q\partial P} & \frac{\partial^{2}\pi }{\partial Q^{2}} \end{array}). \end{array}$$

Next, we apply the principle minor determinants to verify matrix *H*_3×3_ to be negative semi-definite.

$$\begin{array}{c} \begin{array}{cc} & \Delta_{11}=-\frac{P^{*}mb}{nat^{*}}\left(\frac{2b}{n}-b+\epsilon \right)<0\;if\;and\;only\;if\;n\epsilon -\left(n-2\right)b>0,\\ & \Delta_{22}=\frac{m^{2}b}{n^{3}t^{*2}}\left[\frac{\left(a-1)(2b-bn+\epsilon n\right)}{a}-\frac{b}{n}\right]>0\;if\;and\;only\;if\;\epsilon \geq b\;and\;a\geq 2,\\ & \Delta_{33}>0\;if\;and\;only\;if\;\frac{C_{h}\left(an-a-n\right)\left(a-1\right)}{4(MC+\sqrt{\frac{nC_{h}C_{o}}{2m}})}\left(\epsilon -\frac{b}{a}\right)\geq \sqrt{\frac{nmC_{h}}{2C_{o}}\left[\frac{\left(2b-nb+n\epsilon )(a-1\right)}{a}-\frac{n}{b}\right]}. \end{array} \end{array}$$

Therefore, matrix *H*_3×3_ is negative semi-definite, so there exists a unique equilibrium.

$$\begin{array}{c} \begin{array}{cc} & \left(1\right)\frac{\partial Q^{*}}{\partial C_{o}}=\frac{m}{nC_{h}}{\left(\frac{2mC_{o}}{nC_{h}}\right)}^{-\frac{1}{2}}>0,\left(2\right)\frac{\partial Q^{*}}{\partial C_{h}}=-\frac{mC_{o}}{nC_{h}^{2}}{\left(\frac{2mC_{o}}{nC_{h}}\right)}^{-\frac{1}{2}}<0,\left(3\right)\frac{\partial P^{*}}{\partial MC}=\frac{a(n-1)}{an-a-n}>0,\\ & \left(4\right)\frac{\partial P^{*}}{\partial C_{h}}=\frac{a(n-1)}{an-a-n}\frac{1}{2}{\left(\frac{nC_{h}C_{o}}{2m}\right)}^{-\frac{1}{2}}\frac{nC_{o}}{2m}>0,\left(5\right)\frac{\partial P^{*}}{\partial C_{o}}=\frac{a(n-1)}{an-a-n}\frac{1}{2}{\left(\frac{nC_{h}C_{o}}{2m}\right)}^{-\frac{1}{2}}\frac{nC_{h}}{2m}>0,\\ & \left(6\right)\frac{\partial P^{*}}{\partial a}=\left(MC+\sqrt{\frac{nC_{h}C_{o}}{2m}}\right)\frac{\left(-n(n-1)\right)}{{\left(an-a-n\right)}^{2}}<0,\left(7\right)\frac{\partial t^{*}}{\partial MC}=-\frac{t^{*}}{\epsilon P^{*}}\frac{\partial P^{*}}{\partial MC}<0,\\ & \left(8\right)\frac{\partial t^{*}}{\partial C_{h}}=-\frac{t^{*}}{\epsilon P^{*}}\frac{\partial P^{*}}{\partial C_{h}}<0,\left(9\right)\frac{\partial t^{*}}{\partial C_{o}}=-\frac{t^{*}}{\epsilon P^{*}}\frac{\partial P^{*}}{\partial C_{o}}<0,\left(10\right)\frac{\partial t^{*}}{\partial a}=-\frac{t^{*}}{\epsilon P^{*}}\frac{\frac{a\partial P^{*}}{\partial MC}-P^{*}}{a}>0,\\ & \left(11\right)\frac{\partial t^{*}}{\partial b}=-\frac{t^{*}}{b\epsilon }<0,\left(12\right)\frac{\partial t^{*}}{\partial \delta }=\frac{t^{*}}{\epsilon \delta }>0,\left(13\right)\frac{\partial t^{*}}{\partial \epsilon }=\frac{t^{*}}{\epsilon }ln\frac{P^{*}mb}{na\delta \epsilon }+\frac{1}{\epsilon^{2}}>0, \end{array} \end{array}$$

The above findings prove parts (i)–(vi) of Proposition 7.

### Proof of Proposition 8

In a dominated asymmetric oligopoly, we depict the impact of changes in various parameters on three decision variables *t*, *P*, *Q* via satisfying conditions (ii) and (iii) of Lemma 1. Expression (9) takes the following form, setting $t_{i}^{{}^{\prime }}=-t_{i}$ and $P_{i}^{{}^{\prime }}=-P_{i}$:

$$\begin{array}{c} \begin{array}{cc} \pi_{j} & =\left(P_{j}-MC_{j}-\frac{C_{oj}}{Q_{j}}\right)\frac{m\beta_{j}P_{j}^{-a_{j}}t_{j}^{-b_{j}}}{\beta_{j}P_{j}^{-a_{j}}t_{j}^{-b_{j}}+\sum_{i\neq j}^{n}\beta_{i}{\left(-P_{i}^{{}^{\prime }}\right)}^{-a_{i}}{\left(-t_{i}^{{}^{\prime }}\right)}^{-b_{i}}}-\delta_{j}t_{j}^{-\epsilon_{j}}\\ & -\frac{Q_{j}}{2}C_{hj}-F_{j},\;j=1,2,...,n. \end{array} \end{array}$$

Let $G_{j}=\beta_{j}P_{j}^{-a_{j}}t_{j}^{-b_{j}},G_{i}=\beta_{i}{\left(-P_{i}^{{}^{\prime }}\right)}^{-a_{i}}{\left(-t_{i}^{{}^{\prime }}\right)}^{-b_{i}}\left(i\neq j\right).$ Hence,

$$\begin{array}{c} \begin{array}{cc} & \frac{\partial^{2}\pi_{j}}{\partial t_{j}\partial P_{j}}=\frac{mG_{j}}{G_{j}+\sum_{i\neq j}^{n}G_{i}}\left(P_{j}-MC_{j}-\frac{C_{oj}}{Q_{j}}\right)\frac{-a_{j}b_{j}\beta_{j}P_{j}^{a_{j}-1}t_{j}^{-b_{j}-1}\sum_{i\neq j}G_{i}}{{\left(G_{j}+\sum_{i\neq j}G_{i}\right)}^{2}}<0,\\ & \frac{\partial^{2}\pi_{j}}{\partial t_{j}\partial Q_{j}}=\frac{C_{oj}}{Q_{j}^{2}}\frac{-mb_{j}\beta_{j}P_{j}^{-a_{j}}t_{j}^{-b_{j}-1}\sum_{i\neq j}G_{i}}{{\left(G_{j}+\sum_{i\neq j}^{n}G_{i}\right)}^{2}}<0,\frac{\partial^{2}\pi_{j}}{\partial P_{j}\partial Q_{j}}=\frac{mC_{oj}}{Q_{j}^{2}}\frac{-a_{j}P_{j}^{-1}G_{j}\sum_{i\neq j}G_{i}}{{\left(G_{j}+\sum_{i\neq j}G_{i}\right)}^{2}}<0,\\ & \frac{\partial^{2}\pi_{j}}{\partial t_{j}\partial t_{i}^{{}^{\prime }}}=\left(P_{j}-MC_{j}-\frac{C_{oj}}{Q_{j}}\right)\frac{-mb_{j}b_{i}\beta_{j}\beta_{i}P_{j}^{-a_{j}}{\left(-P_{i}^{{}^{\prime }}\right)}^{-a_{i}}t_{j}^{-b_{j}-1}{\left(-t_{i}^{{}^{\prime }}\right)}^{-b_{i}-1}\left(G_{j}-\sum_{i\neq j}G_{i}\right)}{{\left(G_{j}+\sum_{i\neq j}G_{i}\right)}^{3}}<0,\\ & \frac{\partial^{2}\pi_{j}}{\partial t_{j}\partial P_{i}^{{}^{\prime }}}=\left(P_{j}-MC_{j}-\frac{C_{oj}}{Q_{j}}\right)\frac{-ma_{i}b_{j}\beta_{j}\beta_{i}P_{j}^{-a_{j}}{\left(-P_{i}^{{}^{\prime }}\right)}^{-a_{i}-1}t_{j}^{-b_{j}-1}{\left(-t_{i}^{{}^{\prime }}\right)}^{-b_{i}}\left(G_{j}-\sum_{i\neq j}G_{i}\right)}{{\left(G_{j}+\sum_{i\neq j}G_{i}\right)}^{3}}<0,\\ & \frac{\partial^{2}\pi_{j}}{\partial P_{j}\partial t_{i}^{{}^{\prime }}}=\frac{mG_{j}}{G_{j}+\sum_{i\neq j}^{n}G_{i}}\left(P_{j}-MC_{j}-\frac{C_{oj}}{Q_{j}}\right)\frac{-a_{j}b_{i}\beta_{i}P_{j}^{-1}{\left(-P_{i}^{{}^{\prime }}\right)}^{-a_{i}}{\left(-t_{i}^{{}^{\prime }}\right)}^{-b_{i}-1}G_{j}}{{\left(G_{j}+\sum_{i\neq j}^{n}G_{i}\right)}^{2}},\\ & \frac{\partial^{2}\pi_{j}}{\partial P_{j}\partial P_{i}^{{}^{\prime }}}=\frac{mG_{j}}{G_{j}+\sum_{i\neq j}^{n}G_{i}}\left(P_{j}-MC_{j}-\frac{C_{oj}}{Q_{j}}\right)\frac{-a_{j}a_{i}\beta_{i}P_{j}^{-1}{\left(-P_{i}^{{}^{\prime }}\right)}^{-a_{i}-1}{\left(-t_{i}^{{}^{\prime }}\right)}^{-b_{i}}G_{j}}{{\left(G_{j}+\sum_{i\neq j}G_{i}\right)}^{2}},\\ & \frac{\partial^{2}\pi_{j}}{\partial Q_{j}\partial t_{i}^{{}^{\prime }}}=\frac{mC_{oj}}{Q_{j}^{2}}\frac{-b_{i}\beta_{i}{\left(-P_{i}^{{}^{\prime }}\right)}^{-a_{i}}{\left(-t_{i}^{{}^{\prime }}\right)}^{-b_{i}-1}G_{j}}{G_{j}+\sum_{i\neq j}G_{i}}<0,\\ & \frac{\partial^{2}\pi_{j}}{\partial Q_{j}\partial P_{i}^{{}^{\prime }}}=\frac{mC_{oj}}{Q_{j}^{2}}\frac{-a_{i}\beta_{i}{\left(-P_{i}^{{}^{\prime }}\right)}^{-a_{i}-1}{\left(-t_{i}^{{}^{\prime }}\right)}^{-b_{i}}G_{j}}{G_{j}+\sum_{i\neq j}G_{i}}<0,\\ & \frac{\partial^{2}\pi_{j}}{\partial t_{j}\partial Q_{i}}=0,\frac{\partial^{2}\pi_{j}}{\partial P_{j}\partial Q_{i}}=0,\frac{\partial^{2}\pi_{j}}{\partial Q_{j}\partial Q_{i}}=0. \end{array} \end{array}$$

The following proof is similar to that of Proposition 2. For *θ* = *MC*_*j*_, *β*_*j*_, *δ*_*j*_, *ϵ*_*j*_ or *C*_*oj*_, setting *θ*′ = −*θ*, one obtains the results shown in Proposition 8 by applying condition (iv) of Lemma 1. However, for *θ* = *a*_*j*_, *b*_*j*_ or *C*_*hj*_, directly calculating condition (iv) can yield the necessary conclusions.

### Proof of Proposition 9

The proof is similar to Proposition 8. The firm in a non-dominated asymmetric oligopoly only takes decision variables *t*_*j*_ and *P*_*j*_ into account. Setting $t_{j}^{{}^{\prime }}=t_{j}^{-b_{j}},t_{i}^{{}^{\prime }}=-t_{i}^{-b_{i}}$, conditions (ii) and (iii) are satisfied. For the parameters *θ* = *β*_*j*_, *b*_*j*_ *δ*_*j*_ or *ϵ*_*j*_, it is easy to verify condition (iv). However, for parameter *θ* = *a*_*j*_, we set $a_{j}^{{}^{\prime }}=-a_{j}$.
